# The impact of comorbidity status in COVID-19 vaccines effectiveness before and after SARS-CoV-2 omicron variant in northeastern Mexico: a retrospective multi-hospital study

**DOI:** 10.3389/fpubh.2024.1402527

**Published:** 2024-06-12

**Authors:** Maria Elena Camacho-Moll, Viviana Leticia Mata-Tijerina, Carlos Cuauhtémoc Gutiérrez-Salazar, Beatriz Silva-Ramírez, Katia Peñuelas-Urquides, Laura González-Escalante, Brenda Leticia Escobedo-Guajardo, Jorge Eleazar Cruz-Luna, Roberto Corrales-Pérez, Salvador Gómez-García, Mario Bermúdez-de León

**Affiliations:** ^1^Laboratory of Molecular Biology, Northeast Biomedical Research Center, Mexican Social Security Institute, Monterrey, Nuevo Leon, Mexico; ^2^Laboratory of Immunogenetics, Northeast Biomedical Research Center, Mexican Social Security Institute, Monterrey, Nuevo Leon, Mexico; ^3^Center for Mathematical Research (CIMAT), Monterrey, Nuevo León, Mexico; ^4^Laboratory of Molecular Microbiology, Northeast Biomedical Research Center, Mexican Social Security Institute, Monterrey, Nuevo Leon, Mexico; ^5^Laboratory of Molecular Research of Diseases, Northeast Biomedical Research Center, Mexican Social Security Institute, Monterrey, Nuevo Leon, Mexico; ^6^Medical Epidemiological Assistance Coordination of the State of Nuevo Leon, Mexican Social Security Institute, Monterrey, Nuevo Leon, Mexico

**Keywords:** COVID-19, vaccine, comorbidities, hospitalization, mortality

## Abstract

**Introduction:**

The end of the coronavirus disease 2019 (COVID-19) pandemic has been declared by the World Health Organization on May 5, 2023. Several vaccines were developed, and new data is being published about their effectiveness. However, the clinical trials for the vaccines were performed before the Omicron variant appeared and there are population groups where vaccine effectiveness still needs to be tested. The overarching goal of the present study was to analyze the effects of COVID-19 vaccination before and after the Omicron variant in patients considering comorbidities in a population from Nuevo Leon, Mexico.

**Methods:**

Epidemiological COVID-19 data from the Mexican Social Security Institute were collected from 67 hospitals located in northeastern Mexico, from July 2020 to May 2023, and a total of 669,393 cases were compiled, 255,819 reported a SARS-CoV-2 positive reverse transcription quantitative polymerase chain reaction (RT-qPCR) test or a positive COVID-19 antigen rapid test.

**Results:**

Before Omicron (BO, 2020-2021), after 14 days of two doses of COVID-19 vaccine, BNT162b2 and ChAdOx1 vaccines were effective against infection in non-comorbid and all comorbid subgroups, whereas after Omicron (AO, 2022- 2023) there was no significant effectiveness against infection with none of the vaccines. Regarding hospitalization BO, BNT162b2, ChAdOx1, CoronaVac and mRNA-1273 significantly protected non-comorbid patients whereas BNT162b2, ChAdOx1, and mRNA-1273, protected all comorbid subgroups against hospitalization. AO, BNT162b2, ChAdOx1, CoronaVac and mRNA-1273 were effective against hospitalization in non-comorbid patients whereas for most comorbid subgroups BNT162b2, ChAdOx1 and CoronaVac were effective against hospitalization. Non-comorbid patients were protected against death as an outcome of COVID-19 during the BO period with most vaccines whereas a reduction in effectiveness was observed AO with mRNA-1273 vaccines in patients with hypertension, and diabetes mellitus.

**Discussion:**

BO, COVID-19 vaccines were effective against infection, hospitalization, and death whereas AO, COVID-19 vaccines failed to protect the population from COVID-19 infection. A varying effectiveness against hospitalization and death is observed AO.

## 1 Introduction

Coronavirus disease 2019 (COVID-19) has represented a milestone in epidemiological surveillance systems worldwide. Particularly, monitoring the clinical manifestations of the disease, as well as the outcome, have revealed key information about the predisposing factors for complications in different populations, including those observed in Mexicans (1). In previous studies several comorbidities have been associated with an increased risk of severe COVID-19 such as diabetes mellitus (2–5), hypertension (3, 6, 7), chronic kidney disease (3, 8–10), chronic obstructive pulmonary (3, 11–15), rheumatic and autoimmune diseases (16, 17), cancer (18), and obesity (19). Demographic factors such as sex and age have also been associated with severe COVID-19 (1, 20). To reduce the effects led by the causative agent, severe acute respiratory syndrome coronavirus 2 (SARS-CoV-2) (21), several vaccines have been approved to be used during COVID-19 emergency (22). It has been shown that COVID-19 vaccines have an effectiveness of 96-51% against symptomatic COVID-19 disease (23). However, COVID-19 vaccine effectiveness notably decreases after 100 days of immunization, with rates dropping to 26 and 35% for BNT162b2 (Pfizer) following administration of three and four doses (24). This has also been shown in other studies where the effectiveness decreases as time from the vaccination administration elapses specially for prevention of infection (25).

The safety of COVID-19 vaccines has been tested in clinical trials (26–37), and when vaccines were out for the public, several other side effects were reported including anaphylaxis and rare adverse blood coagulation events (38–42). Clinical trials did not include people with comorbidities, pregnant women nor lactating women. The safety of COVID-19 vaccines has been reported in several studies where the most common side effects were pain, redness, or swelling at the injection site and fatigue (43–45). In Mexico, two studies have investigated the side effects of seven COVID-19 vaccines, demonstrating that COVID-19 vaccines are safe, and the benefits of COVID-19 vaccination outweigh the risk of the disease (46, 47).

The vaccination campaign in Mexico started in December 2020, adopting a phased administration approach based on priority groups. The selection of vaccines used depended on to their availability (48). Priority was given to frontline healthcare workers from December 2020 to February 2021, followed by the rest of frontline healthcare workers and people aged 60 and older from February 2021 to April 2021, on a third stage schoolteachers and people aged 50 to 59 were vaccinated from April 2021 to May 2021 followed by people aged 40 to 49 from May 2021 to June 2021, and on a fifth stage which took place in June 2021 the remaining population was vaccinated (48).

Vaccines have been administered to different segments of the population including people with comorbidities, children, and older adults, even though the efficacy and safety clinical trials did not include these groups (27, 32, 36). Although the Centers for Disease Control and Prevention urges people with comorbidities to get vaccinated (49), the evidence that supports protection in comorbid patients is ongoing. For instance, in patients with hypertension there have been studies with controversial results demonstrating a similar vaccine response compared to normotensive patients (50); whereas other studies demonstrated a lower response in hypertensive patients (51). A comparable situation is also observed when analyzing patients with diabetes mellitus with variable vaccine response rates (50, 52). In patients with obesity, it has been shown that the larger the waist circumference there is a lower antibody titer (51). In patients with cancer there is an 90-95% response to vaccine compared to 100% in those without solid tumors (53–55). In the case of patients with non- Hodgkin lymphoma there was a 49% response compared to 98.5% in controls (56). In patients with Chronic lymphocytic leukemia a 52% response was observed compared to 100% in controls (57).

Recently, a study has been published in the northeastern Mexican population demonstrating differences in the effectiveness of four COVID-19 vaccines (58). However, Salinas et al. reported vaccine effectiveness in the studied population controlling with comorbidities, whereas we are describing the effectiveness of the COVID-19 vaccines in non-comorbid and comorbid population (24).

Taboada et al. (59) reported the circulating variants in Mexico from August 5, 2020, to May 31, 2022. The circulating variants during 2020 to 2021 were B.1 (13.5%), B.1.1 (5.2%), B.1.1.222 (10.8%), B.1.1.519 (38.6%), B.1.243 (4.4%), B.1.609 (4.1%), with B.1.1.7 (alpha), P.1 (gamma), B.1.427, B.1.1429 and P.2 representing less than 2% (59). Likewise, B.1.617.2 (Delta) had a prevalence of 87% in August 2021 and since December 2021, the Omicron variant and its sub-variants have been circulating in Mexico (60). COVID-19 incidence can also depend on variants during the different waves as reported in several pre-Omicron studies (61, 62). Based on this evidence, the circulation of Omicron variant was considered as a key factor in our analysis.

In the present study data is divided in two periods of the COVID-19 pandemic which correspond to the period before and after Omicron as it has been shown that in pre-Omicron vaccines, the efficacy against Omicron infection is comparatively lower than that observed for Delta and Alpha strains. Furthermore, the effectiveness of these vaccines in preventing Omicron infection tends to diminish at a faster rate over time (63, 64). According to the WHO, 63% of the total Mexican population has a complete primary series of COVID-19 (65).

The objective of this study was to analyze COVID-19 effectiveness pre- and post-Omicron SARS-CoV-2 variant considering comorbidities and vaccination schemes based on the hypothesis that COVID-19 vaccines effectiveness against infection, hospitalization, and death decreases after Omicron SARS-CoV-2 in patients with and without comorbidities.

## 2 Methods

This protocol was approved by the Ethics and Research Committees of the Mexican Social Security Institute (No. R-2022-1904-118). A retrospective observation study was carried out. Suspected cases for COVID-19 infection from 67 hospitals located in northeastern Mexico were included. These hospitals belonged to the Mexican Social Security Institute. COVID-19 suspected cases dated from August 5, 2020, to May 31, 2022. Inclusion criteria were records with complete information and a reverse transcription quantitative polymerase chain reaction (RT-qPCR) or COVID-19 rapid antigen test result. Those with a positive RT-qPCR test for virus different than COVID-19 were excluded from the analysis. Only records with a positive result for a RT-qPCR or a COVID-19 rapid antigen test were considered as COVID-19 positive. A total of 669,393 cases were included for further analysis constituting a sufficiently large sample size to achieve a statistical power exceeding 95% with a confidence level of 95%.

**Figure 1:**
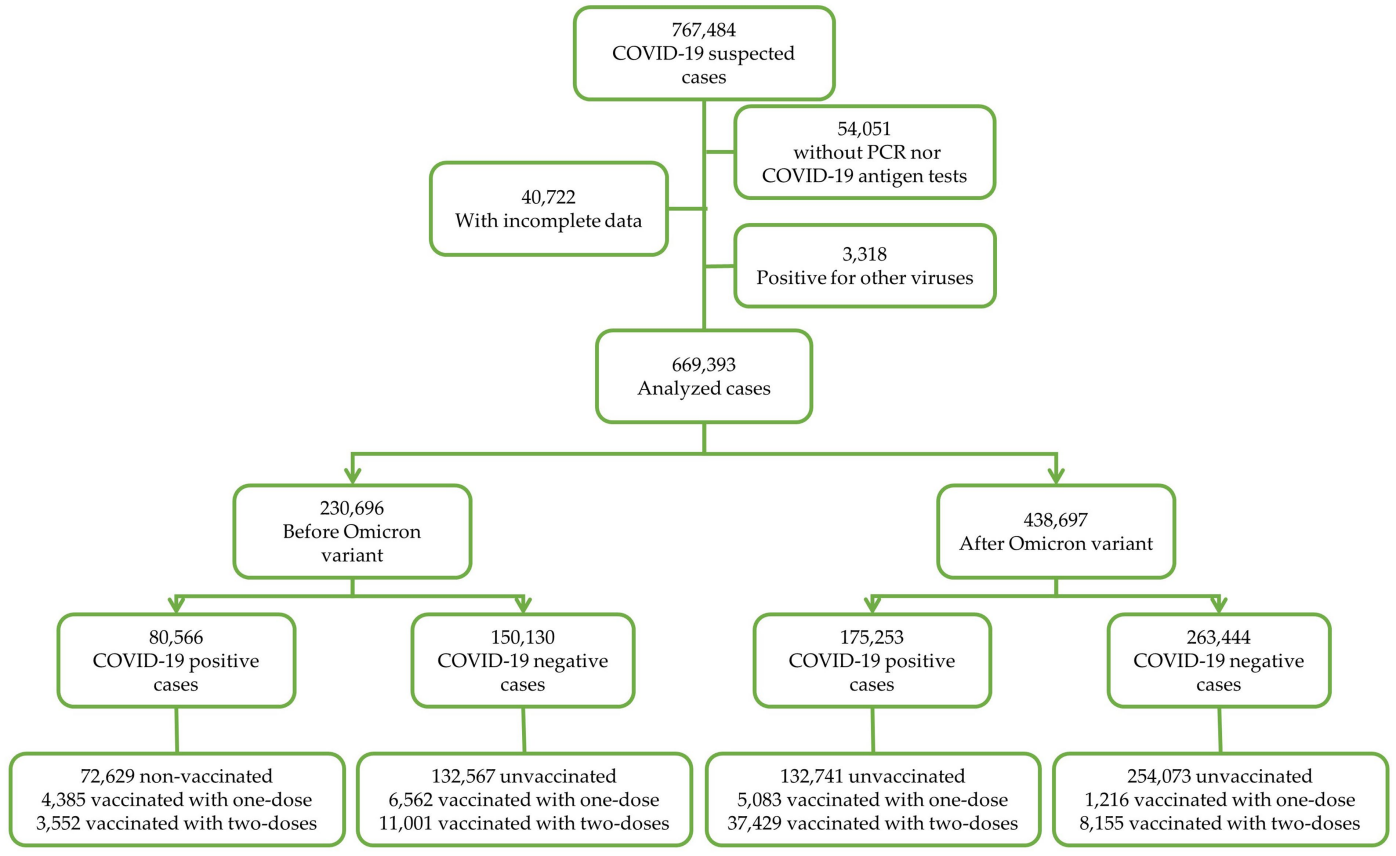
Schematic representation of data depuration.

Data was subgrouped by date where patients who attended the clinic for suspected COVID-19 during 2020-2021, corresponded to the period before Omicron (BO) and those who attended the clinic for suspected COVID-19 during 2022-2023 corresponded to the period after Omicron (AO). From these two groups, data was organized by vaccination status. A schematic representation of data depuration is shown in Figure 1.

### 2.1 Study variables

#### 2.1.1 Vaccine effectiveness

Three main outcomes were analyzed, (a) Infection (yes, no) (b) Hospitalization (yes, no), and (c) Death secondary to COVID-19 complications (yes, no). VE was calculated with the following formula: VE = (1 − OR) × 100% this method is widely used to calculate vaccine effectiveness (25, 66–71).

#### 2.1.2 Type of vaccine

Data included patients vaccinated with the following vaccines: BNT162b2 (Pfizer-BioNTech), ChAdOx1 (AstraZeneca), mRNA-1273 (Moderna), CoronaVac (Sinovac Life Sciences), Gam-COVID-Vac (Gamaleya’s Sputnik V), Ad5-nCoV (CanSinoBIO), and Ad26.CoV2.S (Johnson & Johnson/Janssen), NVX-CoV2373 (Novavax), and BBIBP-CorV (Sinopharm).

#### 2.1.3 Vaccination status

Patients were subgrouped by vaccination status in the following groups: non-vaccinated, one dose with less than 14 days from vaccination to the onset of symptoms, one dose with 14 days of more between vaccination and the onset of symptoms, two doses with less than 14 days from vaccination to the onset of symptoms and two doses with 14 days of more between vaccination and the onset of symptoms.

#### 2.1.4 Comorbidities

Non-comorbid patients represented the individuals with respiratory infection symptoms and tested for COVID-19 (positive or negative result) who did not report any other comorbidity. Only the most common comorbidities were analyzed independently which were obesity, hypertension, and diabetes mellitus. Subgroups with patients with more than one comorbidity were also analyzed, which were patients with two comorbidities and patients with more than two comorbidities.

#### 2.1.5 Control variables

Sex, age, and tobacco smoking were included in the models as control variables.

#### 2.1.6 Statistical analysis

Demographic data distribution was analyzed with chi-square tests. The association of COVID-19 infection, hospitalization and death with vaccination status was performed by stepwise multivariate logistic ordinal regression. From this analysis, different models were performed to calculate odds ratios (OR) and 95% confidence intervals (CI) for each outcome of interest and from these OR (25, 66–71). Stepwise multivariate logistic regression models were adjusted for age, sex, and tobacco smoking, which is described in table footnotes. Tobacco smoking has been previously shown to decrease infection, severity, and death predisposition in COVID-19 positive patient (1). Categorical variables were described in frequencies. For non-categorical variables, means and standard deviation were calculated.

#### 2.1.7 Sources of data

All information was enclosed in the epidemiological data set obtained from the Medical Epidemiological Assistance Coordination of the State of Nuevo Leon, which belongs to the Mexican Social Security Institute. This data set is compiled with information from clinical records.

## 3 Results

### 3.1 Sociodemographic characteristics

Before Omicron (BO), there were more males (52.4%) with COVID-19 compared to females (47.6%). The contrary was observed AO where there were more females infected with COVID-19 compared to males with 55.0 and 45.0%, respectively. In terms of age, the most common affected ages were between 19 and 29 with 30.2% BO and 27.6% AO. The occupation in which the most COVID-19 positive cases were observed were employed/self-employed with 80.1% BO and 85.8% AO. An increased death rate was observed BO with 6.2% compared to 0.7% AO. The most common vaccination status BO and AO was non-vaccinated, and the rates of a full scheme were 6.3% BO and 10.4% AO, respectively. Hospitalization was higher BO, representing 13.3% in COVID-19 positive patients whereas AO hospitalized COVID-19 positive patients represented 2.6%. Most patients were non-comorbid representing 77.4% of the BO population whereas AO this population represented 92.1%. Among the comorbidities identified in the BO group, obesity prevailed at 4.5%, followed by hypertension at 3.3%, and diabetes mellitus at 2.3%. Those with two comorbidities comprised 5.9%, while individuals with more than two comorbidities accounted for 3.0%For the AO period the most common comorbidity was hypertension (1.5%), followed by obesity with 1.2%, and Diabetes Mellitus with 1.0%. Also, AO patients with two comorbidities represented 2.1% whereas patients with more than two comorbidities represented 0.8%. Details of the sociodemographic and other characteristics can be found in Table 1.

**Table 1:** Sociodemographic and other characteristics.

	2020-2021 (*n* = 230,696)				2022-2023 (*n* = 438,697)			
				COVID-19				COVID-19
Sex	Total	Yes	No	Chi-square *p*-value	Total	Yes	No	Chi-square *p*-value
Female	120,042 (52.0)	38,317 (47.6)	81,725 (54.4)	<0.001	225,987 (51.5)	96,336 (55.0)	129,651 (49.2)	<0.001
Male	110,654 (48.0)	42,249 (52.4)	68,405 (45.6)		212,710 (48.5)	78,917 (45.0)	133,793 (50.8)	
*Age*								
0-18	18,236 (7.9)	4,472 (5.6)	13,764 (9.2)	<0.001	52,237 (11.9)	11,333 (6.5)	40,904 (15.59)	<0.001
19-29	74,410 (31.0)	24,364 (30.2)	47,046 (31.3)		129,003 (29.4)	48,387 (27.6)	80,616 (30.6)	
30-39	49,571 (21.5)	18,444 (22.9)	31,127 (20.7)		91,629 (20.9)	40,320 (23.0)	51,309 (19.5)	
40-49	40,239 (17.4)	15,130 (18.8)	25,109 (16.7)		96,091 (17.3)	35,956 (20.5)	40,135 (15.2)	
50-59	27,001 (11.7)	10,084 (12.5)	16,917 (11.3)		54,879 (12.5)	25,942 (14.8)	28,937 (11.0)	
60-69	12,245 (5.3)	4,210 (5.2)	8,035 (5.4)		19,480 (4.4)	7,778 (4.4)	11,702 (4.4)	
>69	11,994 (5.2)	3,862 (4.8)	8,132 (5.4)		15,378 (3.5)	5,537 (3.2)	9,841 (3.7)	
*Occupation*								
Employed/self-employed	173,459 (77.19)	62,733 (80.1)	110,726 (75.4)	<0.001	178,899 (81.7)	144,178 (85.8)	34,721 (68.2)	<0.001
Home maker	20,592 (9.1)	6,925 (8.8)	13,667 (9.3)		13,051 (6.0)	9,004 (5.4)	4,047 (8.0)	
Retired/pensioned	23,602 (10.5)	6,497 (8.3)	17,105 (11.7)		18,389 (8.4)	9,494 (5.7)	8,895 (17.5)	
Student	7,443 (3.3)	2,137 (2.7)	5,306 (3.6)		8,552 (3.9)	5,332 (3.2)	3,220 (6.3)	
*Outcome*								
Recovery	221,690 (96.6)	75,281 (93.8)	146,409 (98.1)	<0.001	340,072 (99.3)	171,517 (99.3)	168,555 (99.3)	0.440
Death	7,832 (3.4)	4,957 (6.2)	2,875 (1.8)		2,447 (0.7)	1,215 (0.7)	1,232 (0.7)	
*COVID-19 vaccination status*								
Non-vaccinated	205,196 (88.9)	72,629 (90.1)	132,567 (88.3)	<0.001	386,814 (88.2)	132,741 (75.7)	254,073 (96.4)	<0.001
One dose	10,947 (4.7)	4,385 (5.4)	6,562 (4.4)		6,299 (1.4)	5,083 (2.9)	1,216 (0.5)	
Two doses or more	14,553 (6.3)	3,552 (4.4)	11,001 (7.3)		45,584 (10.4)	37,429 (21.4)	8,155 (3.1)	
*Hospitalization*								
Yes	31,890 (13.8)	10,732 (13.3)	21,158 (14.1)	<0.001	11,796 (97.3)	4,600 (2.6)	7,196 (2.7)	0.032
No	198,806 (86.2)	69,834 (86.7)	128,972 (85.9)		426,901 (97.3)	170,653 (97.4)	265,248 (97.3)	
Comorbidities		Yes	No			Yes	No	
Non-comorbid	178,536 (77.4)	62,537 (77.6)	115,999 (77.3)	<0.001	403,953 (92.1)	150,455 (85.9)	253,798 (96.2)	<0.001
Obesity	10,418 (4.5)	3,843 (4.8)	,575 (4.4)		5,259 (1.2)	4,237 (2.4)	1,022 (0.4)	
Hypertension	7,674 (3.3)	2,916 (3.6)	4,758 (3.2)		6,676 (1.5)	5,237 (3.0)	1,439 (0.5)	
Diabetes	5,408 (2.3)	2,109 (2.6)	3,299 (2.2)		4,251 (1.0)	3,128 (1.8)	1,123 (0.4)	
Other	3,718 (1.6)	1,052 (1.3)	2,666 (1.8)		2,170 (0.5)	1,482 (0.8)	688 (0.3)	
Asthma	2,498 (1.1)	693 (0.9)	1,805 (1.2)		2,222 (0.5)	1,380 (0.8)	842 (0.3)	
HIV	511 (0.2)	145 (0.2)	366 (0.2)		315 (0.1)	213 (0.1)	102 (0.0)	
Cardiovascular disease	327 (0.1)	93 (0.1)	234 (0.2)		318 (0.1)	177 (0.1)	141 (0.1)	
Renal disease	294 (0.1)	81 (0.1)	213 (0.1)		199 (0.0)	109 (0.1)	90 (0.0)	
COPD	231 (0.1)	58 (0.1)	173 (0.1)		205 (0.0)	104 (0.1)	101 (0.09)	
Immunosuppresion	218 (0.1)	74 (0.1)	144 (0.1)		301 (0.1)	145 (0.1)	156 (0.1)	
Cancer	171 (0.1)	40 (0.0)	131 (0.19)		129 (0.0)	79 (0.0)	50 (0.0)	
Neurolodical disease	86 (0.0)	23 (0.09)	63 (0.0)		74 (0.0)	43 (0.0)	31 (0.0)	
Tuberculoss	48 (0.09)	14 (0.0)	34 (0.0)		32 (0.0)	18 (0.0)	14 (0.0)	
Hepatic disease	41 (0.0)	8 (0.0)	33 (0.0)		30 (0.0)	14 (0.0)	16 (0.0)	
Hemolytic anemia	12 (0.0)	5 (0.0)	7 (0.0)		6 (0.0)	4 (0.0)	2 (0.0)	
Two comorbidities	13,530 (5.9)	4,704 (5.8)	7,826 (5.9)		9,086 (2.1)	6,372 (3.6)	2,714 (1.0)	
More than two comorbidities	6,975 (3.0)	2,171 (2.7)	4,804 (3.2)		3,471 (0.8)	2,056 (1.2)	1,415 (0.5)	

COPD, Chronic Obstructive Pulmonary Disease; HIV, Human Immunodeficiency Virus.

### 3.2 Effectiveness of two doses (more than 14 days) of COVID-19 vaccines against COVID-19 infection

Effectiveness was calculated with the formula (1 − OR) × 100% for nine different vaccines: BNT162b2 (Pfizer), ChAdOx1 (AstraZeneca), CoronaVac (Sinovac), Ad5-nCoV (CansinoBio), mRNA-1273 (Moderna), Ad26.CoV.2 (Johnson & Johnson/Janssen), BBIBP-CorV (Sinopharm), NVX-CoV2373 (Novavax) Gam-COVID-Vac (Gamaleya’s Sputnik V).

During the BO period and for patients with two doses, a significant effectiveness against COVID-19 infection in non-comorbid patients, patients with obesity and patients with hypertension was observed. This effectiveness was consistent across BNT162b2, ChAdOx1, and mRNA-1273 vaccines. For patients with one comorbidity only BNT162b2 and ChAdOx1 were significantly effective against infection. In patients with diabetes mellitus, patients with two comorbidities and patients with more than two comorbidities only BNT162b2 was significantly effective against COVID-19 infection (Table 2).

**Table 2:** Effectiveness of COVID-19 vaccines against infection in patients with two doses.

		BNT162b2	ChAdOx1	CoronaVac	Ad5-nCoV	mRNA-1273
		Effectiveness (1-OR^a^) (%)				
Non-comorbid	BO	55.3*	37.0*	−35.8*	71.1	79.3*
	AO	−797.9*	−969.1*	−1026.4*	−942.9*	−892.8*
One comorbidity	BO	55.2*	36.8*	15.7	26.2	62.6
	AO	−72.0*	−92.4*	−103.6*	−232.4*	−117.0*
Obesity	BO	62.4*	56.6*	5.6	0.0	59.5*
	AO	−11.3	−64.9*	17.1	−276.1	−72*
Hypertension	BO	50.5*	30.9*	15.4	100.0	80.8*
	AO	−73.2*	−47.2*	−92.0*	100.0	−22.1
Diabetes mellitus	BO	67.5*	27.9	24	0.0	63.9
	AO	−89.5*	−64.6*	−183.5*	−76.9	−85.3
2 comorbidities	BO	44.4*	12.5	−22.5	−83.5	46.5
	AO	−92.5*	−100.5*	−144.2*	−79.8	−53.7
>2 comorbidities	BO	60.5*	14.7	0.4	100	78.7
	AO	−57.2*	−30.3*	−59.1*	−7.0	−82.2

*p < 0.005, OR – Odd ratios.

a OR adjusted by sex, age, and tobacco smoking. BO, Before Omicron; AO, After Omicron. Exact *p*-values are described in Supplementary tables – no data available. Other vaccination schemes, 95% CI intervals, *p*-values, and other less represented COVID-19 vaccines can be observed in Supplementary Tables S1, S2 for non-comorbid patients, Supplementary Tables S3, S4 for patients with one comorbidity, Supplementary Tables S5, S6 for patients with obesity, Supplementary Tables S7, S8 for patients with hypertension, Supplementary Tables S9, S10 for patients with Diabetes Mellitus, Supplementary Tables S11, S12 for patients with 2 comorbidities and Supplementary Tables S13, S14 for patients with more than two comorbidities.

During the AO period and in patients with two doses, there was no significant effectiveness against infection with none of the vaccines. Only CoronaVac has a positive value in patients with obesity, however, this was not statistically significant (Table 2).

### 3.3 Effectiveness of one dose (more than 14 days) of COVID-19 vaccines against COVID-19 infection

During the BO period and in non-comorbid patients with one dose, BNT162b2, Ad5-nCoV, which was initially proposed as an only dose full scheme, and mRNA-1273 significantly protected patients from infection with 35, 22 and 61% effectiveness, respectively (Supplementary Table S1), this protection was not observed AO, where non-comorbid patients with an incomplete scheme where not protected from infection (Supplementary Table S2).

In patients with a single comorbidity who received only one dose, both BNT162b2 and mRNA-1273 exhibited significant protection against infection with 52.6 and 71.1% effectiveness, respectively (Supplementary Table S3). However, this protective effect diminished after the emergence of Omicron (Supplementary Table S4). A similar situation was also observed in patients with obesity BO, where BNT162b2 and mRNA-1273 had an effectiveness against infection of 61.5 and 79.1%, respectively (Supplementary Table S5). This protection was lost in AO (Supplementary Table S6). Among patients solely with hypertension, BNT162b2 demonstrated protective effects BO with 51.7% effectiveness, (Supplementary Table S7), which waned AO significantly (Supplementary Table S8).

In patients with diabetes mellitus BO (Supplementary Table S9) and AO (Supplementary Table S10), patients with two comorbidities BO (Supplementary Table S11) and AO (Supplementary Table S12), and patients with more than two comorbidities BO (Supplementary Table S13) and AO (Supplementary Table S14), an incomplete vaccination scheme did not confer significant protection against infection with any of the vaccines.

### 3.4 Effectiveness of two doses (more than 14 days) of COVID-19 vaccines against hospitalization due to COVID-19

Before and after Omicron, BNT162b2, ChAdOx1, CoronaVac and mRNA-1273 significantly protected non-comorbid patients against hospitalization. In patients with one comorbidity also BO and AO, BNT162b2, ChAdOx1, CoronaVac significantly protected patients against hospitalization (Table 3).

**Table 3:** Effectiveness of COVID-19 vaccines against hospitalization in patients with two doses.

		BNT162b2	ChAdOx1	CoronaVac	Ad5-nCoV	mRNA-1273
		Effectiveness (1-OR^a^) (%)				
Non-comorbid	BO	44.5*	66.2*	75.3*	−751.7	37.9*
	AO	44.5*	74.7*	72.8*	−44.8	59.4*
One comorbidity	BO	71.8*	68.2*	71.8*	100.0	62.4
	AO	67*	22.1*	82.3*	−28.2	51.2
Obesity	BO	92.5*	68.6*	53.9	100.0	100.0
	AO	3.3	−68.9	34.6	100.0	−80.0
hypertension	BO	69.3*	79.4*	97.28*	-	100.0
	AO	47.6*	3.6	70.5*	−390.3	−75.3
Diabetes mellitus	BO	79.3*	69*	70*	−	100.0
	AO	69.2*	11.2	73.9*	−219.5	30.0
2 comorbidities	BO	70.9*	63.8*	71.6*	39.7	100.0
	AO	69*	32.7*	51.6*	100.0	78.5
>2 comorbidities	BO	50.4*	65.8*	73.7*	−	100.0
	AO	65.1*	23.4	55.4*	−139	100.0

*p < 0.005, OR, Odd ratios.

a OR adjusted by sex, age, and tobacco smoking. BO, Before Omicron; AO, After Omicron. Exact *p*-values are described in Supplementary tables – no data available. Other vaccination schemes, 95% CI intervals, *p*-values, and other less represented COVID-19 vaccines can be observed in Supplementary Tables S1, S2 for non-comorbid patients, Supplementary Tables S3, S4 for patients with one comorbidity, Supplementary Tables S5, S6 for patients with obesity, Supplementary Tables S7, S8 for patients with hypertension, Supplementary Tables S9, S10 for patients with Diabetes Mellitus, Supplementary Tables S11, S12 for patients with 2 comorbidities and Supplementary Tables S13, S14 for patients with more than two comorbidities.

In patients with one comorbidity BO, Ad5-nCoV was 100% effective against hospitalization, whereas this was nor observed AO (−28.2%) (Table 3).

In patients with obesity and BO, BNT162b2, ChAdOx1, Ad5nCoV and mRNA-1273 significantly protected patients against hospitalization. After Omicron 100% effectiveness was observed with Ad5-nCov (Table 3).

In the BO period, in patients with hypertension and Diabetes Mellitus, BNT162b2, ChAdOx1, CoronaVac, and mRNA-1273 significantly protected patients against hospitalization. During the AO period, BNT162b2 and CoronaVac continued to provide protection against hospitalization for hypertensive and diabetic patients (Table 3).

BNT162b2, ChAdOx1, and CoronaVac demonstrated significant protective effects against hospitalization in patients with two or more comorbidities, BO and AO. Notably, mRNA-1273 exhibited 100% effectiveness before Omicron, while Ad5-nCoV showed the same level of effectiveness after Omicron (Table 3). Similarly, for patients with more than two comorbidities, BNT162b2, ChAdOx1, and CoronaVac remained significantly effective against hospitalization both BO and AO, with mRNA-1273 exhibiting 100% effectiveness in both periods (Table 3).

### 3.5 Effectiveness of one dose (more than 14 days) of COVID-19 vaccines against hospitalization due to COVID-19

During the BO period Omicron BNT162b2, ChAdOx1, CoronaVac, Ad5-nCoV and mRNA-1273 significantly protect non-comorbid patients against hospitalization with 47.7, 74.2, 63.6, 72.5 and 78.1% effectiveness, respectively (Supplementary Table S1). During the AO period this protection was reduced and failed to reach significance. However, 100% effectiveness against hospitalization is observed with CoronaVac and Ad5-nCoV (Supplementary Table S2). In patients with one comorbidity (Supplementary Table S3), obesity (Supplementary Table S5) and hypertension (Supplementary Table S7), ChAdOx1 and mRNA-1273 significantly protected patients from hospitalization BO. The protection provided by ChAdOx1 is lost AO. However, with mRNA-1273 the effectiveness is 100% (Supplementary Tables S4, S6, S8). In patients with obesity and hypertension AO, Ad4-nCov exhibited a 100% effectiveness protecting against hospitalization (Supplementary Tables S6, S8). For individuals with diabetes mellitus (Supplementary Table S9), ChAdOx1, Ad5-nCoV, and mRNA-1273 provided significant protection against hospitalization BO, whereas AO, only CoronaVac, Ad5-nCoV, and mRNA-1273 demonstrated a 100% effectiveness against hospitalization (Supplementary Table S10). In patients with two comorbidities, protection against hospitalization was observed with ChAdOx1 and Ad5-nCoV BO (Supplementary Table S11). AO, CoronaVac and Ad5-nCoV exhibited 100% effectiveness (Supplementary Table S12). For patients with two comorbidities BO (Supplementary Table S13), mRNA-1273 demonstrated 100% protection. AO, (Supplementary Table S14), both mRNA-1273 and CoronaVac maintained 100% effectiveness against hospitalization.

### 3.6 Effectiveness of two doses (more than 14 days) of COVID-19 vaccines against death as an outcome of COVID-19

During the BO period, in non-comorbid patients, BNT162b2, ChAdOx1, CoronaVac, and Ad5-nCoV, were significantly effective against death, whereas AO, BNT162b2, ChAdOx1, Ad5-nCoV and mRNA-1273 protected COVID-19 positive patients against death as an outcome of COVID-19 (Table 4).

**Table 4:** Effectiveness of COVID-19 vaccines against death as an outcome of COVID-19 infection in patients with two doses.

		BNT162b2	ChAdOx1	CoronaVac	Ad5-nCoV	mRNA-1273
		Effectiveness (1-OR^a^) (%)				
Non-comorbid	BO	71.9*	70.3*	70.2*	100.0	30.9
	AO	62.2*	61.1*	53.0	100.0	100.0
One comorbidity	BO	72.9*	74.8*	67.5*	100.0	100.0
	AO	68.1*	42.8*	81.7*	100.0	−70.0
Obesity	BO	100.0	86.8*	78.9	100.0	100.0
	AO	10.6	−230.7	100.0	100.0	100.0
Hypertension	BO	64.8*	76.8*	40.9	100.0	100.0
	AO	63.2	33.0	100.0	100.0	−260.3
Diabetes mellitus	BO	90.7*	77.9*	100.0	–	100.0
	AO	69.8	77.5*	68.4	100.0	−197.8
2 comorbidities	BO	73.8*	58.3*	60.4*	100.0	100.0
	AO	49.4*	32.6	24.4	100.0	100.0
>2 comorbidities	BO	69.7*	40.2*	53.6	−	100.0
	AO	63.0*	54.6*	89.4*	100.0	100.0

*p < 0.005, OR – Odd ratios.

a OR adjusted by sex, age, and tobacco smoking. BO, Before Omicron; AO, After Omicron. Exact *p*-values are described in Supplementary tables – no data available. Other vaccination schemes, 95% CI intervals, *p*-values, and other less represented COVID-19 vaccines can be observed in Supplementary Tables S1, S2 for non-comorbid patients, Supplementary Tables S3, S4 for patients with one comorbidity, Supplementary Tables S5, S6 for patients with obesity, Supplementary Tables S7, S8 for patients with hypertension, Supplementary Tables S9, S10 for patients with Diabetes Mellitus, Supplementary Tables S11, S12 for patients with 2 comorbidities and Supplementary Tables S13, S14 for patients with more than two comorbidities.

In patients with one comorbidity, BO, and AO all the COVID-10 vaccines shown in Table 4 were effective against death as an outcome of COVID-19 infection, AO, only mRNA-1273 vaccine failed to reach significance (Table 4).

In patients with obesity ChadOx1, BNT162b2, Ad5-nCoV and mRNA-1273 significantly protected patients against death BO. In the AO period, CoronaVac, Ad5-nCoV and mRNA-1273 showed a 100% effectiveness against death (Table 4).

In patients with hypertension, BO, BNT162b22, and ChAdOx1 were significantly effective against death. A 100% effectiveness was observed with mRNA-1273. After Omicron a 100% effectiveness was observed with CoronaVac and Ad5-nCoV (Table 4).

During the BO period, in patients with diabetes mellitus, BNT162b2, ChAdOx1 CoronaVac, and mRNA-1273 were effective against death whereas AO, only ChAdOx1 was significantly effective against death and a 100% effectiveness was observed with Ad5-nCoV (Table 4).

In patients with two comorbidities, BO, all the vaccines shown in Table 4 were effective against death due to COVID-19, whereas after Omicron ChAdOx1 and CoronaVac failed to reach significance (Table 4).

In patients with more than two comorbidities, BO, BNT162b2 and ChAdOx1 were significantly effective against death, with a 100% effectiveness observed with mRNA-1273, whereas AO a protection against death was observed with BNT162b2, ChAdOx1, CoronaVac, Ad5-nCoV, and mRNA-1273 (Table 4).

### 3.7 Effectiveness of one dose (more than 14 days) of COVID-19 vaccines against death as an outcome of COVID-19

During the BO period ChadOx1 protected non-comorbid patients from death (Supplementary Table S1) whereas AO BNT162b2, CoronaVac, Ad5-nCoV and mRNA-1273 showed a 100% effectiveness (Supplementary Table S2). Before Omicron in patients with one comorbidity ChAdOx1 significantly protected patients against death and 100% effectiveness was observed with Ad5-nCov and mRNA-1273 (Supplementary Table S3) whereas AO, 100 % effectiveness was observed with CoronaVac, Ad5-nCov and mRNA-1273 (Supplementary Table S4).

During de BO period patients with obesity were significantly protected with ChAdOx1 and 100% effectiveness was shown with Ad5-nCoV and mRNA-1273 (Supplementary Table S5). After Omicron BNT162b2, ChAdOx1, CoronaVac, Ad5nCoV and mRNA1273 vaccines demonstrated 100% effectiveness against death (Supplementary Table S6).

In patients with hypertension, BO, 100% effectiveness was shown with CoronaVac, Ad5-nCoV and mRNA-1273 (Supplementary Table S7), while AO all vaccines significantly protected against death except for ChAdOx1 (Supplementary Table S8).

In patients with diabetes mellitus, a 100% protection was observed with ChAdOx1, Ad5-nCoV and mRNA1273 (Supplementary Table S9), whereas AO, the effectiveness remained with CoronaVac, Ad5-nCoV and mRNA1273 (Supplementary Table S10).

In patients with two comorbidities ChAdOx1 significantly protected patients against death BO (Supplementary Table S11), whereas a 100% protection against death was observed with CoronaVac and Ad5-nCoV and mRNA1273 AO (Supplementary Table S12).

In patients with more than two doses BO (Supplementary Table S13) and AO (Supplementary Table S14) 100% effectiveness was shown with CoronaVac and mRNA-1273.

## 4 Discussion

The current study describes the effectiveness of COVID-19 vaccines against infection, hospitalization, and death in comorbid and non-comorbid patients from Nuevo León, Mexico, with special attention to those patients with comorbidities such as obesity, diabetes mellitus, and hypertension. Furthermore, this analysis describes the results BO and AO demonstrating a notorious decrease in COVID-19 vaccines effectiveness against infection in the AO period.

This study included in the analysis age, sex, and tobacco smoking as controls in the model given that it has been shown that in people older than 60 years of age, full vaccination, consisting of at least 2 doses of COVID-19 vaccine, effectively protected against pneumonia and risk of severe COVID-19 caused by a specific omicron variant (72). Also, vaccination has been found to reduce hospitalization rates by 37%, and the admission to emergency room by 24% in vaccinated versus unvaccinated patients (73).

Data was split by periods BO (2020-2021) and AO (2022-2023) as it has been shown that pre-Omicron vaccines are less effective in protecting against infection with SARS-CoV-2 Omicron variant compared to Delta and Alpha infections (63).

### 4.1 Before Omicron

The first observational studies regarding the immunogenicity of COVID-19 vaccines in relation to some comorbidities were reported during the period BO (50–52, 74–82). It is imperative to mention that among the comorbidities that affect the Mexican population, obesity is a major public health concern, as in 2018, 22% of the children were obese and in people between 30 and 59 years of age, 46% of women were obese and35% of men were obese (83). Obese patients have an increased risk of hospitalization due to COVID-19 particularly those under 60 years of age with a body mass index (BMI) of 35 kg/m^2^ or higher, who exhibit an increased susceptibility to intensive care unit (ICU) admission. Notably, patients with a BMI of 40 kg/m^2^ or higher have an increased predisposition to mortality due to COVID-19 (28). As for vaccine effectiveness in obese patients, there have been some controversies as it has been shown in some studies that BMI has no impact on BNT162b2 vaccine effectiveness (84) whereas others have shown lower antibody titers correlate with increase waist circumference (85). In the present study we show that two doses (more than 14 days) of BNT162b2, ChAdOx1 and mRNA-1273 effectively protect patients against infection BO. A significant protection against hospitalization was observed in patients with obesity BO with BNT162b2, ChAdOx1 and Ad5-nCoV also with two doses (more than 14 days). It is important to mention that Ad5-nCoV is a single dose vaccine, therefore patients with one dose are considered with a full scheme (86). Before Omicron BNT162b2, ChAdOx1, Ad5-nCoV protected obese patients against death due to COVID-19.

Hypertension is another comorbidity that affects around 20.7% of the Mexican population older than 20 years old (87). Hypertension has been associated with severe COVID-19, and the prevalence of hypertension is higher in patients admitted to ICU (88). During this period a study demonstrated a significant reduction in vaccine response in hypertense patients (51). Watanabe and colleagues demonstrated that hypertense patients were protected against death when two doses have been administered (51). Moreover, it has been shown that there is an association between increased waist circumference and hypertension with lower antibody titers (51). We do observe protection against infection, hospitalization, and death in hypertense patients. However, not with all tested vaccines and not at the same level. Before Omicron protection against infection ranged from 30.9% for ChAdOx1 to 100% for Ad5-nCoV.Also, in patients with hypertension, BNT162b2 and CoronaVac significantly protected hypertense patients against hospitalization whereas CoronaVac and Ad5-nCov protected to a 100% against death.

Diabetes mellitus is also on the top 5 public health concerns in Mexico; in 2020, 151,019 persons died of diabetes mellitus (89), this would be 12 out of 10,000 people, which is the highest rate in the last 10 years, and this rate increases with age (89). Diabetes mellitus is also the third cause of death just below COVID-19, and heart diseases (89). It has been shown that in patients with diabetes mellitus, there is an increased expression of ACE-2, the main receptor of SARS-CoV-2, in the lung and other organs (90), which situates diabetic patients at increased risk. Paggi et al. (82) demonstrated, with 420 COVID-19 positive patients, that the vaccinated group had a higher Charlson comorbidity index compared to the unvaccinated group (82), where the most frequent diseases were cardiovascular diseases, pulmonary diseases, renal diseases, diabetes mellitus, dementia, cancer, and hematological diseases. No difference in terms of in-hospital mortality, was observed. Therefore, it was imperative to study the effects of the COVID-19 vaccines in these comorbid patients. In the case of diabetic patients, there are two studies demonstrating controversial results in diabetic patients. One demonstrating no differences in the response when compared to healthy adults (50), and the other demonstrated a response reduction (52). Coexisting conditions such as diabetes, obesity, hypertension, and age have also been shown to affect antibody generation by anti-COVID-19 vaccines (76, 77).

Different studies have also shown a reduction in vaccine response for chronic obstructive pulmonary disease (COPD) patients (81), hepatic diseases (74) chronic diseases as a group and cardiovascular disease (52), and patients with celiac disease (80).

Recently, a report has been published with the effectiveness of two doses of three COVID-19 vaccines against infection, hospitalization, and severity with an effectiveness against infection of 74.5, 33.2, −2.9% for BNT162b2, ChAdOx1 and Sinovac, respectively (58). Compared to our results, in non-comorbid patients we observe 55.3, 37.0, −35.8%, for BNT162b2, ChAdOx1 and Sinovac, respectively on a BO period, which are similar results. Salinas et al. (58) did not report COVID-19 vaccine effectiveness by comorbidity and only a 6-month period before Omicron is being analyzed.

### 4.2 After Omicron

In patients with hypertension, no difference has been reported compared with healthy patients when vaccinated with BNT162b2 or Sinopharm COVID-19 vaccines (50). In the present study there was no protection observed against infection AO in hypertensive patients. In immunocompromised patients a 48% reduction in antibody titers compared to healthy individuals has been reported (78).

As for other reports investigating vaccination response in comorbid patients, in a study in a Japanese population with 1,041 hospitalized and previously vaccinated individuals, it was reported that in vaccinated patients ≥60 years of age with diabetes and hypertension there is a lower adjusted OR to trigger severe COVID-19 (75). In patients with obesity, CoronaVac and Ad5nCov protected patients against death. Ad5-nCoV also protected comorbid patients against hospitalization with 100% effectiveness.

A strategy that could be used to address the decrease of vaccine effectiveness AO could be booster campaigns, or co-administration of COVID-19 vaccination along with influenza annual booster, this latter is already established immunization program in Mexico (91). Around 90% of the Mexican population gets vaccinated against influenza every year (92). The acceptance and safety of the co-administration of COVID-19 and Influenza vaccines have been studied elsewhere where it has been demonstrated that among 2,740 healthcare workers approximately 60% accepted the co-administration of the vaccines (93) and a complementary study demonstrated its co-administration is safe (45).

## 5 Conclusion

Our results suggest that some vaccines lost effectiveness in the AO period, especially in patients with comorbidities. Further research should be invested in developing vaccines with higher effectiveness against circulating variants and intended for immunocompromised patients such as those with Diabetes Mellitus who are also at increased risk of severe COVID-19. A follow-up should be carried out in vaccinated patients to test the medium-term outcomes such as 6 months after vaccination as it has been demonstrated that female gender, young age, past infection, two vaccine doses, and m-RNA and heterologous vaccination predicted higher antibody levels at 6 months (94).

### 5.1 Limitations

Antibody titration was not performed; therefore, vaccine response rates are not available. A previous COVID-19 infection might have played a role in patient response to COVID-19 disease. More information for less represented vaccines is required such as Ad26.CoV2.S, BBIBP-CorV, NVX-CoV2373 and Gam-COVID-Vac.

## Data availability statement

The original contributions presented in the study are included in the article/Supplementary materials, further inquiries can be directed to the corresponding author.

## Ethics statement

The study was conducted in accordance with the Declaration of Helsinki and approved by the Institutional Review Board of the Mexican Social Security Institute (protocol code: R-2022-1904-118, 31/10/2022). The studies were conducted in accordance with the local legislation and institutional requirements. Written informed consent for participation was not required from the participants or the participants’ legal guardians/next of kin because only epidemiological data was used.

## Author contributions

MC-M: Conceptualization, Data curation, Formal analysis, Investigation, Methodology, Supervision, Validation, Visualization, Writing – original draft. VM-T: Conceptualization, Data curation, Formal analysis, Investigation, Visualization, Writing – review & editing. CG-S: Data curation, Formal analysis, Methodology, Validation, Writing – review & editing. BS-R: Conceptualization, Investigation, Methodology, Supervision, Visualization, Writing – review & editing. KP-U: Conceptualization, Investigation, Visualization, Writing – review & editing. LG-E: Conceptualization, Investigation, Visualization, Writing – review & editing. BE-G: Conceptualization, Investigation, Methodology, Visualization, Writing – review & editing. JC-L: Data curation, Resources, Writing – review & editing. RC-P: Data curation, Resources, Writing – review & editing. SG-G: Data curation, Resources, Writing – review & editing. MB-DL: Writing – review & editing, Resources, Supervision, Visualization.
