# Supplementary material for: The impact of comorbidity status in COVID-19 vaccines effectiveness before and after SARS-CoV-2 omicron variant in northeastern Mexico: a retrospective multi-hospital study
Source: Front Public Health. 2024 Jun 12;12:1402527. doi: 10.3389/fpubh.2024.1402527 (PMC11199416; doi:10.3389/fpubh.2024.1402527)
Supplement: Supplementary file 1 [file Data_Sheet_1.ZIP › Table S10.docx]

**Table S10.** COVID-19 vaccines effectiveness in patients with Diabetes Mellitus after Omicron.

| **Diabetes Mellitus, after Omicron** | | | | | | | | | | | | | |
| --- | --- | --- | --- | --- | --- | --- | --- | --- | --- | --- | --- | --- | --- |
|  |  | COVID-19 infection | | | | Hospitalization | | | | Death | | | |
|  | Total | Yes | No | Effectiveness (95%CI) (Adjusted 1 – OR) | *p*-value | Yes | No | Effectiveness (95%CI) (Adjusted 1 – OR) | *p*-value | Yes | No | Effectiveness (95%CI) (Adjusted 1 – OR) | *p*-value |
| **BNT162b2 (Pfizer)** |  |  |  |  |  |  |  |  |  |  |  |  |  |
| No vaccine | 2,821 (94.2) | 1,964 (82.1) | 857 (89.6) | Ref. |  | 141 (91.6) | 1,823 (81.5) | Ref. |  | 51 (91.1) | 1,889 (81.7) | Ref. |  |
| 1st dose ≥14 days | 33 (1.0) | 26 (1.1) | 7 (0.7) | -57.5% (-264.5%,31.9%) | 0.289 | 4 (2.6) | 22 (1.0) | -240.9% (-985.5%,-7.1%) | 0.038 | 2 (3.6) | 24 (1.0) | -414.6% (-2410.8%,-5.5%) | 0.049 |
| 2nd dose 0-13 days | 1 (0.0) | 1 (0.0) | 0 (0.0) | 0% | - | 0 (0.0) | 1 (0.0) | 100% | - | 0 (0.0) | 1 (0.0) | 100% | - |
| 2nd dose ≥14 days | 494 (14.8) | 401 (16.8) | 93 (9.7) | -89.5% (-140.8%,-49.1%) | <0.001 | 9 (5.8) | 392 (17.5) | 69.2% (38.4%,84.6%) | 0.001 | 3 (5.4) | 397 (17.2) | 69.8% (0.9%,90.8%) | 0.055 |
| **ChAdOx1 (AstraZeneca)** |  |  |  |  |  |  |  |  |  |  |  |  |  |
| No vaccine | 2,821 (82.6) | 1,964 (80.6) | 857 (87.5) | Ref. |  | 141 (85.5) | 1,823 (80.2) | Ref. |  | 51 (94.4) | 1,889 (80.2) | Ref. |  |
| 1st dose ≥14 days | 52 (1.5) | 42 (1.7) | 10 (1.0) | -79% (-259%,10.8%) | 0.101 | 1 (0.6) | 41 (1.8) | 53.8% (-247.8%,93.9%) | 0.454 | 1 (1.9) | 41 (1.7) | -68.5% (-1256.5%,79.1%) | 0.624 |
| 2nd dose 0-13 days | 1 (0.0) | 1 (0.0) | 0 (0.0) | 0% | - | 0 (0.0) | 1 (0.0) | 100% | - | 0 (0.0) | 1 (0.0) | 100% | - |
| 2nd dose ≥14 days | 542 (15.9) | 460 (17.6) | 112 (11.4) | -64.6% (-105.9%,-31.7%) | <0.001 | 23 (13.9) | 407 (17.9) | 11.2% (-41%,44.9%) | 0.599 | 2 (3.7) | 425 (18.0) | 77.5% (4.9%,94.7%) | 0.043 |
| **CoronaVac (Sinovac)** |  |  |  |  |  |  |  |  |  |  |  |  |  |
| No vaccine | 2,821 (93.2) | 1,694 (91.7) | 857 (96.8) | Ref. |  | 141 (97.9) | 1,823 (91.2) | Ref. |  | 51 (98.1) | 1,889 (91.4) | Ref. |  |
| 1st dose ≥14 days | 10 (0.3) | 8 (0.4) | 2 (0.2) | -77.8% (-742.2%,62.5%) | 0.468 | 0 (0.0) | 8 (0.4) | 100% | - | 0 (0.0) | 8 (0.4) | 100% | - |
| 2nd dose 0-13 days | 1 (0.0) | 1 (0.0) | 0 (0.0) | 100% | - | 0 (0.0) | 1 (0.1) | 100% | - | 0 (0.0) | 1 (0.0) | 100% | - |
| 2nd dose ≥14 days | 195 (6.4) | 169 (7.9) | 26 (2.9) | -183.5% (-331.9%,-86.1%) | <0.001 | 3 (2.1) | 166 (8.3) | 73.9% (16.7%,91.8%) | 0.023 | 1 (1.9) | 168 (8.1) | 68.4% (-133.5%,95.7%) | 0.259 |
| **Ad5-nCoV (CanSinoBIO)** |  |  |  |  |  |  |  |  |  |  |  |  |  |
| No vaccine | 2,821 (99.3) | 1,694 (99.0) | 857 (99.8) | Ref. |  | 141 (99.3) | 1,823 (99.0) | Ref. |  | 51 (100.0) | 1,889 (99.1) | Ref. |  |
| 1st dose ≥14 days | 11 (0.4) | 11 (0.6) | 0 (0.0) | 0% | - | 0 (0.0) | 11 (0.6) | 100% | - | 0 (0.0) | 11 (0.6) | 100% | - |
| 2nd dose ≥14 days | 10 (0.4) | 8 (0.4) | 2 (0.2) | -76.9% (-738.9%,62.7%) | 0.472 | 1 (0.7) | 7 (0.4) | -219.5% (-2681.5%,63.3%) | 0.293 | 0 (0.0) | 7 (0.4) | 100% | - |
| **mRNA-1273 (Moderna)** |  |  |  |  |  |  |  |  |  |  |  |  |  |
| No vaccine | 2,821 (97.5) | 1,964 (97.1) | 857 (98.5) | Ref. |  | 141 (99.3) | 1,823 (97.0) | Ref. |  | 51 (98.1) | 1,889 (97.1) | Ref. |  |
| 1st dose ≥14 days | 13 (0.4) | 11 (0.5) | 2 (0.2) | -126.8% (-926.6%,49.9%) | 0.288 | 0 (0.0) | 11 (0.6) | 100% | - | 0 (0.0) | 11 (0.6) | 100% | - |
| 2nd dose ≥14 days | 58 (2.0) | 47 (2.3) | 11 (1.3) | -85.3% (-262.3%,5.3%) | 0.072 | 1 (0.7) | 46 (2.4) | 30% (-445.5%,91%) | 0.734 | 1 (1.9) | 45 (2.3) | -197.8% (-2504%,65.9%) | 0.324 |
| **Ad26.CoV2.S (Johnson & Johnson/Janssen)** |  |  |  |  |  |  |  |  |  |  |  |  |  |
| No vaccine | 2,821 (99.99) | 1,964 (99.8) | 857 (100.0) | Ref. |  | 141 (100.0) | 1,823 (99.8) | Ref. |  | 51 (100.0) | 1,889 (99.8) | Ref. |  |
| 1st dose ≥14 days | 2 (0.1) | 2 (0.1) | 0 (0.0) | 0% | - | 0 (0.0) | 2 (0.1) | 100% | - | 0 (0.0) | 2 (0.1) | 100% | - |
| 2nd dose ≥14 days | 1 (0.0) | 1 (0.1) | 0 (0.0) | 0% | - | 0 (0.0) | 1 (0.1) | 100% | - | 0 (0.0) | 1 (0.1) | 100% | - |
| **BBIBP-CorV (Sinopharm)** |  |  |  |  |  |  |  |  |  |  |  |  |  |
| No vaccine | 2,821 (99.8) | 1,964 (99.8) | 857 (99.9) | Ref. |  | 141 (100.0) | 1,823 (99.8) | Ref. |  | 51 (100.0) | 1,889 (99.8) | Ref. |  |
| 1st dose ≥14 days | 1 (0.0) | 1 (0.1) | 0 (0.0) | 0% | - | 0 (0.0) | 1 (0.1) | 100% | - | 0 (0.0) | 1 (0.1) | 100% | - |
| 2nd dose ≥14 days | 4 (0.1) | 3 (0.2) | 1 (0.1) | -27.1% (-1125.5%,86.8%) | 0.835 | 0 (0.0) | 3 (0.2) | 100% | - | 0 (0.0) | 3 (0.2) | 100% | - |

OR – Odd ratios, OR adjusted for sex, age, and tobacco smoking.
