# Supplementary material for: The impact of comorbidity status in COVID-19 vaccines effectiveness before and after SARS-CoV-2 omicron variant in northeastern Mexico: a retrospective multi-hospital study
Source: Front Public Health. 2024 Jun 12;12:1402527. doi: 10.3389/fpubh.2024.1402527 (PMC11199416; doi:10.3389/fpubh.2024.1402527)
Supplement: Supplementary file 1 [file Data_Sheet_1.ZIP › Table S4.docx]

**Table S4.** COVID-19 vaccines effectiveness in patients with one comorbidity after Omicron.

| **With one comorbidity, after Omicron** | | | | | | | | | | | | | |
| --- | --- | --- | --- | --- | --- | --- | --- | --- | --- | --- | --- | --- | --- |
|  |  | COVID-19 infection | | | | Hospitalization | | | | Death | | | |
|  | Total | Yes | No | Effectiveness (95%CI) (Adjusted 1 – OR) | *p*-value | Yes | No | Effectiveness (95%CI) (Adjusted 1 – OR) | *p*-value | Yes | No | Effectiveness (95%CI) (Adjusted 1 – OR) | *p*-value |
| **BNT162b2 (Pfizer)** |  |  |  |  |  |  |  |  |  |  |  |  |  |
| No vaccine | 14,090 (82.2) | 9,813 (80.1) | 4,277 (87.3) | Ref. |  | 574 (91.8) | 9,239 (79.5) | Ref. |  | 200 (93.5) | 9,508 (79.8) | Ref. |  |
| 1st dose 0-13 days | 4 (0.0) | 1 (0.0) | 3 (0.1) | 80.2% (-90.9%,97.9%) | 0.161 | 0 (0.0) | 1 (0.0) | 100% | - | 0 (0.0) | 1 (0.0) | 100% | - |
| 1st dose ≥14 days | 209 81.29 | 169 81.4) | 40 (0.8) | -98% (-180.4%,-39.8%) | <0.001 | 9 (1.4) | 160 (1.4) | -33.6% (-166.6%,33.1%) | 0.412 | 1 (0.9) | 166 (1.4) | -23.1% (-416.8%,70.7%) | 0.776 |
| 2nd dose 0-13 days | 13 (0.1) | 9 (0.1) | 4 (0.1) | -6.9% (-249.5%,67.3%) | 0.912 | 0 (0.0) | 9 (0.1) | 100% | - | 0 (0.0) | 9 (0.1) | 100% | - |
| 2nd dose ≥14 days | 2830 (16.5) | 2,255 (18.4) | 575 11.7) | -72% (-89.8%,-55.8%) | <0.001 | 42 (6.7) | 2,213 (19.0) | 67% (54.6%,76%) | <0.001 | 12 (5.6) | 2,234 (18.7) | 68.1% (42.2%,82.4%) | <0.001 |
| **ChAdOx1 (AstraZeneca)** |  |  |  |  |  |  |  |  |  |  |  |  |  |
| No vaccine | 14,090 (81.2) | 9,813 (78.7) | 4,277 (87.4) | Ref. |  | 574 (83.9) | 9,239 (78.4) | Ref. |  | 200 (88.5) | 9,508 (78.5) | Ref. |  |
| 1st dose 0-13 days | 7 (0.0) | 3 (0.0) | 4 (0.1) | 66.9% (-48%,92.6%) | 0.148 | 0 (0.0) | 3 (0.0) | 100% |  | 0 (0.0) | 3 (0.0) | 100% | - |
| 1st dose ≥14 days | 372 (2.1) | 293 (2.4) | 79 (1.6) | -69.3% (-117.7%,-31.6%) | <0.001 | 10 (1.5) | 283 (2.4) | 12.7% (-67%,54.4%) | 0.682 | 3 (1.3) | 289 (2.4) | -13% (-272.6%,65.7%) | 0.841 |
| 2nd dose 0-13 days | 19 (0.1) | 15 (0.1) | 4 (0.1) | -71% (-416.7%,43.4%) | 0.341 | 1 (0.1) | 14 (0.1) | -137.5% (-1760.7%,69.7%) | 0.41 | 0 (0.0) | 15 (0.1) | 100% | - |
| 2nd dose ≥14 days | 2,871 (16.5) | 2,343 (18.8) | 528 (10.8) | -92.4% (-112.9%,-73.9%) | <0.001 | 99 (14.5) | 2,244 (19.0) | 22.1% (2.6%,37.8%) | 0.028 | 23 (10.2) | 2,301 (19.0) | 42.8% (10.4%,63.5%) | 0.015 |
| **CoronaVac (Sinovac)** |  |  |  |  |  |  |  |  |  |  |  |  |  |
| No vaccine | 41,090 (93.8) | 9,813 (92.6) | 4,277 (96.7) | Ref. |  | 574 (98.3) | 9,239 (92.3) | Ref. |  | 200 (99.0) | 9,508 (92.5) | Ref. |  |
| 1st dose ≥14 days | 74 (0.5) | 68 (0.6) | 6 (0.1) | -381.5% (-1011.1%,-108.6%) | <0.001 | 2 (0.3) | 66 (0.7) | 48.3% (-114%,87.5%) | 0.363 | 0 (0.0) | 68 (0.7) | 100% | - |
| 2nd dose 0-13 days | 2 (0.0) | 2 (0.0) | 0 (0.0) | 0% | - | 0 (0.0) | 2 (0.09 | 100% | - | 0 (0.0) | 2 (0.0) | 100% | - |
| 2nd dose ≥14 days | 853 (5.7) | 711 (6.7) | 142 (3.2) | -103.6% (-144.9%,-69.3%) | <0.001 | 8 (1.4) | 703 (7.0) | 82.3% (64.3%,91.3%) | <0.001 | 2 (1.0) | 706 (6.9) | 81.7% (25.8%,95.5%) | 0.017 |
| **Ad5-nCoV (CanSinoBIO)** |  |  |  |  |  |  |  |  |  |  |  |  |  |
| No vaccine | 14,090 (99.1) | 9,813 (98.9) | 4,277 (99.6) | Ref. |  | 574 (99.5) | 9,239 (98.9) | Ref. |  | 200 (100.0) | 9,508 (98.9) | Ref. |  |
| 1st dose ≥14 days | 83 (0.6) | 71 (0.7) | 12 (0.3) | -167.1% (-393.5%,-44.5%) | 0.002 | 1 (0.2) | 70 (0.7) | 66.4% (-143.7%,95.4%) | 0.28 | 0 (0.0) | 71 (0.7) | 100% | - |
| 2nd dose 0-13 days | 1 (0.0) | 1 (0.0) | 0 (0.0) | 0% |  | 0 (0.0) | 1 (0.0) | 100% | - | 0 (0.0) | 1 (0.0) | 100% | - |
| 2nd dose ≥14 days | 42 (0.3) | 37 (0.4) | 5 (0.1) | -232.4% (-747.9%,-30.3%) | 0.012 | 2 (0.3) | 35 (0.4) | -28.2% (-441.4%,67%) | 0.736 | 0 (0.0) | 36 (0.4) | 100% | - |
| **mRNA-1273 (Moderna)** |  |  |  |  |  |  |  |  |  |  |  |  |  |
| No vaccine | 14,090 (95.6) | 9,813 (94.9) | 4,277 (97.2) | Ref. |  | 574 (99.0) | 9,239 (94.6) | Ref. |  | 200 (98.5) | 9,508 (94.8) | Ref. |  |
| 1st dose 0-13 days | 1 (0.0) | 0 (0.0) | 1 (0.0) | 100% |  | 0 (0.0) | 0 (0.0) | 100% | - | 0 (0.0) | 0 (0.0) | - | - |
| 1st dose ≥14 days | 89 (0.6) | 74 (0.7) | 15 (0.3) | -142.1% (-322.8%,-38.7%) | 0.002 | 0 (0.0) | 74 (0.8) | 100% | - | 0 (0.0) | 74 (0.7) | 100% | - |
| 2nd dose 0-13 days | 2 (0.0) | 1 (0.0) | 1 (0.0) | 49.1% (-716.4%,96.8%) | 0.633 | 0 (0.0) | 1 (0.0) | 100% | - | 0 (0.0) | 1 (0.0) | 100% | - |
| 2nd dose ≥14 days | 561 (3.8) | 455 (4.4) | 106 (2.4) | -117% (-169.5%,-74.7%) | <0.001 | 6 (1.0) | 449 (4.6) | 51.2% (-11%,78.5%) | 0.087 | 3 (1.5) | 451 (4.5) | -70% (-469.4%,49.2%) | 0.39 |
| **Ad26.CoV2.S (Johnson & Johnson/Janssen)** |  |  |  |  |  |  |  |  |  |  |  |  |  |
| No vaccine | 14,2090 (99.7) | 9,813 (99.7) | 4,277 (99.7) | Ref. |  | 574 (100.0) | 9,239 (99.7) | Ref. |  | 200 (100.0) | 9,508 (99.7) | Ref. |  |
| 1st dose ≥14 days | 31 (0.2) | 23 (0.2) | 8 (0.2) | -45.1% (-225.4%,35.3%) | 0.366 | 0 (0.0) | 23 (0.2) | 100% | - | 0 (0.0) | 23 (0.2) | 100% | - |
| 2nd dose ≥14 days | 10 (0.1) | 7 (0.1) | 3 (0.1) | -13.3% (-340.1%,70.9%) | 0.857 | 0 (0.0) | 7 (0.1) | 100% | - | 0 (0.0) | 7 (0.1) | 100% | - |
| **BBIBP-CorV (Sinopharm)** |  |  |  |  |  |  |  |  |  |  |  |  |  |
| No vaccine | 14,090 (99.9) | 9,813 (99.9) | 4,277 (99.9) | Ref. |  | 574 (99.9) | 9,239 (99.9) | Ref. |  | 200 (100.0) | 9,508 (99.9) | Ref. |  |
| 1st dose ≥14 days | 2(0.0) | 1 (0.0) | 1 (0.0) | 56.3% (-610.9%,97.3%) | 0.561 | 0 (0.0) | 1 (0.0) | 100% | - | 0 (0.0) | 1 (0.0) | 100% | - |
| 2nd dose ≥14 days | 11 (0.1) | 9 (0.1) | 2 (0.0) | -64% (-660.6%,64.6%) | 0.527 | 1 (0.2) | 8 (0.1) | -36.2% (-1024.8%,83.5%) | 0.774 | 0 (0.0) | 9 (0.1) | 100% | - |
| **NVX-CoV2373 (Novavax)** |  |  |  |  |  |  |  |  |  |  |  |  |  |
| No vaccine | 14,090 (100.0) | 9,813 (99.9) | 4,277 (100.0) | Ref. |  | 574 (99.8) | 9,239 (99.9) | Ref. |  | 200 (100.0) | 9,508 (99.9) | Ref. |  |
| 2nd dose ≥14 days | 6 (0.0) | 6 (0.1) | 0 (0.0) | 0% | - | 1 (0.2) | 5 (0.1) | -252.8% (-3027.5%,60.2%) | 0.257 | 0 (0.0) | 6 (0.1) | 100% | - |
| **Gam-COVID-Vac (Gamaleya’s Sputnik V)** |  |  |  |  |  |  |  |  |  |  |  |  |  |
| No vaccine | 14,090 (100.0) | 9,813 (100.0) | 4,277 (100.0) | Ref. |  | 574 (99.8) | 9,239 (100.0) | Ref. |  | 200 (100.0) | 8,508 (100.0) | Ref. |  |
| 2nd dose ≥14 days | 4 (0.0) | 3 (0.0) | 1 (0.0) | -41.4% (-1262.5%,85.3%) | 0.764 | 1 (0.2) | 2 (0.0) | -657.6% (-8957.1%,36.6%) | 0.11 | 0 (0.0) | 2 (0.0) | 100% | - |

OR – Odd ratios, OR adjusted for sex, age, and tobacco smoking.
