# Supplementary material for: The impact of comorbidity status in COVID-19 vaccines effectiveness before and after SARS-CoV-2 omicron variant in northeastern Mexico: a retrospective multi-hospital study
Source: Front Public Health. 2024 Jun 12;12:1402527. doi: 10.3389/fpubh.2024.1402527 (PMC11199416; doi:10.3389/fpubh.2024.1402527)
Supplement: Supplementary file 1 [file Data_Sheet_1.ZIP › Table S5.docx]

**Table S5.** COVID-19 vaccines effectiveness in patients with obesity before Omicron.

| **Obesity, before Omicron** | | | | | | | | | | | | | | |
| --- | --- | --- | --- | --- | --- | --- | --- | --- | --- | --- | --- | --- | --- | --- |
|  |  | COVID-19 infection | | | | Hospitalization | | | | Death | | | | |
|  | Total | Yes | No | Effectiveness (95%CI) (Adjusted 1 – OR) | *p*-value | Yes | No | Effectiveness (95%CI) (Adjusted 1 – OR) | *p*-value | Yes | No | Effectiveness (95%CI) (Adjusted 1 – OR) | *p*-value |  |
| **BNT162b2 (Pfizer)** |  |  |  |  |  |  |  |  |  |  |  |  |  |  |
| No vaccine | 8,663 (93.5) | 3,290 (96.3) | 5,373 (91.8) | Ref. |  | 415 (98.8) | 2,875 (95.9) | Ref. |  | 196 (99.5) | 3,079 (96.0) | Ref. |  |  |
| 1st dose 0-13 days | 56 (0.6) | 24 (0.7) | 32 (0.5) | -18.4% (-102.1%,30.6%) | 0.536 | 1 (0.2) | 23 (0.8) | 65.5% (-158.9%,95.4%) | 0.301 | 0 (0.0) | 24 (0.7) | 100% | - |  |
| 1st dose ≥14 days | 102 (1.1) | 20 (0.6) | 82 (1.4) | 61.5% (63.9%,76.5%) | <0.001 | 3 (0.7) | 17 (0.6) | -5.3% (-297.9%,70.8%) | 0.937 | 1 (0.5) | 19 (0.6) | 32.9% (-453.9%,91.9%) | 0.711 |  |
| 2nd dose 0-13 days | 25 (0.3) | 4 (0.1) | 21 (0.4) | 67% (3.3%,88.7%) | 0.043 | 0 (0.0) | 4 (0.1) | 100% | - | 0 (0.0) | 4 (0.1) | 100% | - |  |
| 2nd dose ≥14 days | 424 (4.6) | 80 (2.3) | 344 (5.9) | 62.4% (51.8%,70.7%) | <0.001 | 1 (0.2) | 79 (2.6) | 92.5% (45.6%,99%) | 0.01 | 0 (0.0) | 80 (2.5) | 100% | - |  |
| **ChAdOx1 (AstraZeneca)** |  |  |  |  |  |  |  |  |  |  |  |  |  |  |
| No vaccine | 8,663 (92.1) | 3,290 (91.5) | 5,373 (92.4) | Ref. |  | 415 (95.4) | 2,875 (91.0) | Ref. |  | 196 (96.6) | 3,079 (91.2) | Ref. |  |  |
| 1st dose 0-13 days | 111 (1.2) | 64 (1.8) | 47 (0.8) | -119.5% (-221.4%,-49.8%) | <0.001 | 8 (1.8) | 56 (1.89 | -16% (-148.6%,45.9%) | 0.704 | 4 (2.0) | 60 (1.8) | -46.1% (-314.8%,48.6%) | 0.477 |  |
| 1st dose ≥14 days | 393 (4.2) | 184 (5.1) | 209 (3.6) | -42.4% (-74.6%,-16%) | 0.001 | 7 (1.6) | 177 (5.6) | 72.4% (40.6%,87.2%) | 0.001 | 2 (1.0) | 181 (5.4) | 79.9% (17.7%,98.1%) | 0.026 |  |
| 2nd dose 0-13 days | 44 (0.5) | 11 (0.3) | 33 (0.6) | 46.2% (-7.1%,73%) | 0.078 | 1 (0.2) | 10 (0.3) | 65.3% (-182.3%,95.7%) | 0.322 | 0 (0.0) | 11 (0.3) | 100% | - |  |
| 2nd dose ≥14 days | 196 (2.1) | 45 (1.3) | 151 (2.6) | 56.6% (39%,69.1%) | <0.001 | 4 (0.9) | 41 (1.3) | 68.6% (7.9%,89.3%) | 0.035 | 1 (0.5) | 44 (1.3) | 86.8% (-0.5%,98.3%) | 0.047 |  |
| **CoronaVac (Sinovac)** |  |  |  |  |  |  |  |  |  |  |  |  |  |  |
| No vaccine | 8,633 (98.1) | 3,290 (97.9) | 5 ,373 (98.2) | Ref. |  | 415 (98.1) | 2,875 (97.8) | Ref. |  | 196 (98.5) | 3,079 (97.8) | Ref. |  |  |
| 1st dose 0-13 days | 16 (0.2) | 7 (0.2) | 9 (0.2) | -21.3% (-227.4%,55.1%) | 0.704 | 1 (0.2) | 6 (0.2) | -44.7% (-1128.3%,83%) | 0.735 | 1 (0.5) | 6 (0.2) | -326.4% (-3599.3%,50.9%) | 0.188 |  |
| 1st dose ≥14 days | 56 (0.6) | 24 (0.7) | 32 (0.6) | -8.7% (-85.9%,36.4%) | 0.759 | 2 (0.5) | 22 (0.7) | 60.7% (-69.2%,90.9%) | 0.21 | 1 (0.5) | 23 (0.7) | 57.4% (-0.6%,94.3%) | 0.407 |  |
| 2nd dose 0-13 days | 15 (0.2) | 7 (0.2) | 8 (0.1) | -24% (-244.2%,55.3%) | 0.679 | 1 (0.2) | 6 (0.2) | 41.4% (-402.1%,93.2%) | 0.626 | 0 (0.0) | 7 (0.2) | 100% | - |  |
| 2nd dose ≥14 days | 85 (1.0) | 34 (1.0) | 51 (0.9) | 5.6% (-46.7%,39.3%) | 0.796 | 4 (0.9) | 30 (1.0) | 53.9% (-33.5%,84.1%) | 0.154 | 1 (0.5) | 33 (1.0) | 78.9% (-57.2%,97.2%) | 0.129 |  |
| **Ad5-nCoV (CanSinoBIO)** |  |  |  |  |  |  |  |  |  |  |  |  |  |  |
| No vaccine | 8,663 (99.4) | 3,290 (99.5) | 5,373 (99.4) | Ref. |  | 415 (99.8) | 2,875 (99.4) | Ref. |  | 196 (100.0) | 3,079 (99.4) | Ref. |  |  |
| 1st dose 0-13 days | 3 (0.0) | 2 (0.1) | 1 (0.0) | -236.4% (-3734.4%,70.5%) | 0.328 | 0 (0.0) | 2 (0.1) | 100% | 100% | 0 (0.0) | 2 (0.1) | 100% | - |  |
| 1st dose ≥14 days | 49 (0.6) | 15 (0.5) | 34 (0.6) | 25.9% (-36.9%,59.9%) | 0.339 | 1 (0.2) | 14 (0.5) | 57.7% (-230.3%,94.7%) | 0.412 | 0 (0.0) | 15 (0.5) | 100% | - |  |
| 2nd dose ≥14 days | 1 (0.0) | 1 (0.0) | 0 (0.0) | 0% | - | 0 (0.0) | 1 (0.0) | 100% | - | 0 (0.0) | 1 (0.0) | 100% | - |  |
| **mRNA-1273 (Moderna)** |  |  |  |  |  |  |  |  |  |  |  |  |  |  |
| No vaccine | 8,663 (98.1) | 3,290 (99.2) | 5,373 (97.4) | Ref. |  | 415 (100.0) | 2,875 (99.1) | Ref. |  | 196 (100.0) | 3,079 (99.1) | Ref. |  |  |
| 1st dose 0-13 days | 12 (0.1) | 6 (0.2) | 6 (0.1) | -71% (-436%,45.4%) | 0.57 | 0 (0.0) | 6 (0.2) | 100% | 0.999 | 0 (0.0) | 6 (0.2) | 100% | - |  |
| 1st dose ≥14 days | 58 (0.7) | 6 (0.2) | 52 (0.9) | 79.1% (51.1%,91%) | <0.001 | 0 (0.0) | 0 (0.0) | 100% | - | 0 (0.0) | 6 (0.2) | 100% | - |  |
| 2nd dose 0-13 days | 16 (0.29 | 0 (0.0) | 16 (0.3) | 100% | - | 0 (0.0) | 0 (0.0) | - | - | 0 (0.0) | 0 (0.0) |  | - |  |
| 2nd dose ≥14 days | 83 (0.9) | 15 (0.5) | 68 (1.2) | 59.5% (28.9%,77%) | 0.002 | 0 (0.0) | 15 (0.5) | 100% | - | 0 (0.0) | 15 (0.5) | 100% | - |  |
| **Ad26.CoV2.S (Johnson & Johnson/Janssen)** |  |  |  |  |  |  |  |  |  |  |  |  |  |  |
| No vaccine | 8,663 (99.9) | 3,290 (99.9) | 5,373 (99.9) | Ref. |  | 415 | 2,875 (99.9) | Ref. |  | 196 (100.0) | 3,079 (99.9) | Ref. |  |  |
| 1st dose 0-13 days | 1 (0.0) | 0 (0.0) | 1 (0.0) | 100% | - | 0 (0.0) | 0 (0.0) | 100% | - | 0 (0.0) | 0 (0.0) |  | - |  |
| 1st dose ≥14 days | 6 (0.1) | 3 (0.1) | 3 (0.1) | -43.6% (-624.5%,71.6%) | 0.662 | 0 (0.0) | 3 (0.1) | 100% | - | 0 (0.0) | 3 (0.1) | 100% | - |  |
| 2nd dose ≥14 days | 1 (0.0) | 1 (0.01) | 0 (0.0) | 0% | - | 0 (0.0) | 1 (0.0) | 100% | - | 0 (0.0) | 1 (0.0) | 100% | - |  |

OR – Odd ratios, OR adjusted for sex, age, and tobacco smoking.
