# Supplementary material for: The impact of comorbidity status in COVID-19 vaccines effectiveness before and after SARS-CoV-2 omicron variant in northeastern Mexico: a retrospective multi-hospital study
Source: Front Public Health. 2024 Jun 12;12:1402527. doi: 10.3389/fpubh.2024.1402527 (PMC11199416; doi:10.3389/fpubh.2024.1402527)
Supplement: Supplementary file 1 [file Data_Sheet_1.ZIP › Table S14.docx]

**Table S14.** COVID-19 vaccines effectiveness in patients with more than two comorbidities after Omicron.

| **More than two comorbidities, after Omicron** | | | | | | | | | | | | | |
| --- | --- | --- | --- | --- | --- | --- | --- | --- | --- | --- | --- | --- | --- |
|  |  | COVID-19 infection | | | | Hospitalization | | | | Death | | | |
|  | Total | Yes | No | Effectiveness (95%CI) (Adjusted 1 – OR) | *p*-value | Yes | No | Effectiveness (95%CI) (Adjusted 1 – OR) | *p*-value | Yes | No | Effectiveness (95%CI) (Adjusted 1 – OR) | *p*-value |
| **BNT162b2 (Pfizer)** |  |  |  |  |  |  |  |  |  |  |  |  |  |
| No vaccine | 2,285 (84.0) | 1,284 (81.6) | 1,001 (87.3) | Ref. |  | 332 (91.0) | 952 (78.8) | Ref. |  | 134 (91.29 | 1,114 (80.5) | Ref. |  |
| 1st dose 0-13 days | 1 (0.0) | 0 (0.0) | 1 (0.1) | 100% | - | 0 (0.0) | 0 (0.0) | - | - | 0 (0.0) | 0 (0.0) | - | - |
| 1st dose ≥14 days | 31 (1.1) | 18 (1.1) | 13 (1.1) | -0.8% (-107.7%,51.1%) | 0.972 | 4 (1.1) | 14 (1.2) | -0.6% (-228.8%,69.2%) | 0.998 | 3 (2.0) | 14 (1.0) | -143.3% (-806.5%,34.7%) | 0.185 |
| 2nd dose ≥14 days | 403 (14.8) | 271 (17.2) | 132 (11.5) | -57.2% (-96.9%,-25.5%) | <0.001 | 29 (7.9) | 242 (20.0) | 65.1% (46.6%,77.2%) | <0.001 | 10 (6.8) | 255 (18.4) | 63% (27.8%,81.1%) | 0.004 |
| **ChAdOx1 (AstraZeneca)** |  |  |  |  |  |  |  |  |  |  |  |  |  |
| No vaccine | 2,285 (80.7) | 1,284 (78.9) | 1,001 (83.2) | Ref. |  | 332 (82.8) | 952 (77.7) | Ref. |  | 134 (89.3) | 1,114 (77.8) | Ref. |  |
| 1st dose 0-13 days | 3 (0.1) | 0 (0.0) | 3 (0.2) | 100% | - | 0 (0.0) | 0 (0.0) | - | - | 0 (0.0) | 0 (0.0) | - | - |
| 1st dose ≥14 days | 49 (1.7) | 36 (2.2) | 13 (1.1) | -107.2% (-295.7%,-8.5%) | 0.027 | 9 (2.2) | 27 (2.2) | -12.8% (-160.8%,51.2%) | 0.778 | 1 (0.7) | 34 (2.4) | 74.6% (-94.6%,96.7%) | 0.187 |
| 2nd dose 0-13 days | 5 (0.2) | 2 (0.1) | 3 (0.2) | 47.3% (-223.2%,91.4%) | 0.489 | 1 (0.2) | 1 (0.1) | -176% (-9265.8%,91.9%) | 0.572 | 1 (0.7) | 1 (0.1) | -768.2% (-28367.3%,73.5%) | 0.225 |
| 2nd dose ≥14 days | 488 (17.2) | 305 (18.7) | 183 (15.2) | -30.3% (-59.8%,-6.3%) | 0.011 | 59 (14.7) | 246 (20.1) | 23.4% (-7.2%,45.3%) | 0.12 | 14 (9.3) | 283 (19.8) | 54.6% (18.5%,74.7%) | 0.008 |
| **CoronaVac (Sinovac)** |  |  |  |  |  |  |  |  |  |  |  |  |  |
| No vaccine | 2,285 (93.0) | 1,285 (91.6) | 1,001 (94.8) | Ref. |  | 332 (96.5) | 952 (90.1) | Ref. |  | 134 (99.3) | 1,114(90.8) | Ref. |  |
| 1st dose ≥14 days | 9 (0.4) | 6 (0.4) | 3 (0.3) | -46.3% (-487.5%,63.6%) | 0.592 | 0 (0.0) | 6 (0.6) | 100% | - | 0 (0.0) | 6 (0.5) | 100% | - |
| 2nd dose ≥14 days | 163 (6.6) | 111 (7.9) | 52 (4.9) | -59.1% (-123.8%,-13.1%) | 0.008 | 12 (3.5) | 99 (9.4) | 55.4% (15.5%,76.4%) | 0.013 | 1 (0.7) | 107 (8.7) | 89.4% (22.5%,98.5%) | 0.027 |
| **Ad5-nCoV (CanSinoBIO)** |  |  |  |  |  |  |  |  |  |  |  |  |  |
| No vaccine | 2,285(99.5) | 1,284 (99.4) | 1,001 (99.6) | Ref. |  | 332 (98.8) | 952 (99.6) | Ref. |  | 134 (99.3) | 1,114 (99.4) | Ref. |  |
| 1st dose ≥14 days | 7 (0.3) | 5 (0.4) | 2 (0.2) | -73.9% (-805.4%,66.6%) | 0.511 | 3 (0.9) | 2 (0.2) | -877.7% (-7597.7%,-24.2%) | 0.03 | 1 (0.79 | 4 (0.4) | -207.4% (-3171.2%,71.1%) | 0.352 |
| 2nd dose ≥14 days | 5 (0.2) | 3 (0.2) | 2 (0.2) | -7.0% (-543%,82.2%) | 0.941 | 1 (0.3) | 2 (0.2) | -139% (-2577.7%,78.7%) | 0.48 | 0 (0.0) | 3 (0.3) | 100% | - |
| **mRNA-1273 (Moderna)** |  |  |  |  |  |  |  |  |  |  |  |  |  |
| No vaccine | 2,285 (99.3) | 1,284 (99.2) | 1,001 899.5) | Ref. |  | 332 (100.0) | 952 (98.9) | Ref. |  | 134 (100.0) | 1,114 (99.0) | Ref. |  |
| 1st dose ≥14 days | 4 (0.2) | 2 (0.2) | 2 (0.2) | 33.8% (-375.4%,90.8%) | 0.682 | 0 (0.0) | 2 (0.2) | 100% | - | 0 (0.0) | 2 (0.2) | 100% | - |
| 2nd dose ≥14 days | 12 (0.5) | 9 (0.7) | 3 (0.3) | -82.2% (-579.7%,51.2%) | 0.372 | 0 (0.0) | 9 (0.9) | 100% | - | 0 (0.0) | 9 (0.8) | 100% | - |
| **BBIBP-CorV (Sinopharm)** |  |  |  |  |  |  |  |  |  |  |  |  |  |
| No vaccine | 2,285 (99.9) | 1,284 (99.8) | 1,001 (99.9) | Ref. |  | 332 (99.7) | 952 (99.9) | Ref. |  | 134 (99.3) | 1,114 (99.9) | Ref. |  |
| 1st dose ≥14 days | 1(0.0) | 0 (0.0) | 1 (0.1) | 100% | - | 0 (0.0) | 0 (0.0) | - | - | 0 (0.0) | 0 (0.0) | - | - |
| 2nd dose ≥14 days | 2 (0.1) | 2 (0.2) | 0 (0.0) | 0% | - | 1 (0.3) | 1 (0.1) | -343.5% (-7702.8%,74.8%) | 0.309 | 1 (0.7) | 1 (0.1) | -1235.8% (-23462.2%,24.3%) | 0.077 |
| **NVX-CoV2373 (Novavax)** |  |  |  |  |  |  |  |  |  |  |  |  |  |
| No vaccine | 2,285 (99.9) | 1,284 (99.8) | 1,001 (100.0) | Ref. |  | 332 (99.7) | 952 (99.9) | Ref. |  | 134 (100.0) | 1,114 (99.8) | Ref. |  |
| 1st dose ≥14 days | 1 (0.0) | 1 (0.1) | 0 (0.0) | 0% | - | 1 (0.3) | 0 (0.0) | 0% | - | 0 (0.0) | 1 (0.1) | 100% | - |
| 2nd dose ≥14 days | 1 (0.0) | 1 (0.1) | 0 (0.0) | 0% | - | 0 (0.0) | 1 (0.1) | 100% | - | 0 (0.0) | 1 (0.1) | 100% | - |

OR – Odd ratios, OR adjusted for sex, age, and tobacco smoking.
