# Supplementary material for: The impact of comorbidity status in COVID-19 vaccines effectiveness before and after SARS-CoV-2 omicron variant in northeastern Mexico: a retrospective multi-hospital study
Source: Front Public Health. 2024 Jun 12;12:1402527. doi: 10.3389/fpubh.2024.1402527 (PMC11199416; doi:10.3389/fpubh.2024.1402527)
Supplement: Supplementary file 1 [file Data_Sheet_1.ZIP › Table S6.docx]

**Table S6.** COVID-19 vaccines effectiveness in patients with obesity after Omicron.

| **Obesity, after Omicron** | | | | | | | | | | | | | |
| --- | --- | --- | --- | --- | --- | --- | --- | --- | --- | --- | --- | --- | --- |
|  |  | COVID-19 infection | | | | Hospitalization | | | | Death | | | |
|  | Total | Yes | No | Effectiveness (95%CI) (Adjusted 1 – OR) | *p*-value | Yes | No | Effectiveness (95%CI) (Adjusted 1 – OR) | *p*-value | Yes | No | Effectiveness (95%CI) (Adjusted 1 – OR) | *p*-value |
| **BNT162b2 (Pfizer)** |  |  |  |  |  |  |  |  |  |  |  |  |  |
| No vaccine | 2,899 (76.5) | 2,278 (75.9) | 621 (78.5) | Ref. |  | 19 (79.2) | 2,259 (75.9) | Ref. |  | 4 (80.0) | 2,247 (75.8) | Ref. |  |
| 1st dose ≥14 days | 62 (1.6) | 56 (1.9) | 6 (0.8) | -162.6% (-513.6%,-12.4%) | 0.026 | 0 (0.0) | 56 (1.9) | 100% | - | 0 (0.0) | 56 (1.9) | 100% | - |
| 2nd dose 0-13 days | 2 (0.1) | 2 (0.1) | 0 (0.0) | 0% | - | 0 (0.0) | 2 (0.1) | 100% | - | 0 (0.0) | 2 (0.1) | 100% | - |
| 2nd dose ≥14 days | 828 (21.8) | 664 (22.1) | 164 (20.7) | -11.3% (-35%,8.3%) | 0.278 | 5 (20.8) | 659 (22.1) | 3.3% (-162.5%,64.4%) | 0.947 | 1 (20.0) | 658 (22.2) | 10.5% (-960.1%,92.4%) | 0.930 |
| **ChAdOx1 (AstraZeneca)** |  |  |  |  |  |  |  |  |  |  |  |  |  |
| No vaccine | 2,899 (75.3) | 2,278 (73.8) | 621 (81.4) | Ref. |  | 19 (65.5) | 2,259 (73.9) | Ref. |  | 4 (57.1) | 2,247 (73.8) | Ref. |  |
| 1st dose 0-13 days | 2 (0.0) | 0 (0.0) | 2 (0.3) | 100% | - | 0 (0.0) | 0 (0.0) | 100% | - | 0 (0.0) | 0 (0.0) | 100% | - |
| 1st dose ≥14 days | 116 (3.0) | 93 (3.0) | 23 (3.0) | -16.5% (-85.7%,26.9%) | 0.521 | 1 (3.4) | 92 (3.0) | -114% (-1566.8%,72.5%) | 0.468 | 0 (0.0) | 93 (3.1) | 100% | - |
| 2nd dose 0-13 days | 5 (0.1) | 5 (0.2) | 0 (0.0) | 0% | - | 0 (0.0) | 5 (0.2) | 100% | - | 0 (0.0) | 5 (0.2) | 100% | - |
| 2nd dose ≥14 days | 826 (21.5) | 709 (23.0) | 117 (15.3) | -64.9% (-104.6%,-33%) | <0.001 | 9 (31.0) | 700 (22.9) | -68.9% (-284.5%,25.2%) | 0.206 | 3 (42.9) | 700 (23.0) | -230.7% (-1688.8%,38.8%) | 0.165 |
| **CoronaVac (Sinovac)** |  |  |  |  |  |  |  |  |  |  |  |  |  |
| No vaccine | 2,899 (94.6) | 2,278 (94.5) | 621 (94.8) | Ref. |  | 19 (90.5) | 2,259 (94.6) | Ref. |  | 4 (100.0) | 2,247 (94.5) | Ref. |  |
| 1st dose ≥14 days | 17 (0.6) | 16 (0.7) | 1 (0.2) | -328.7% (-3145.8%,43.4%) | 0.159 | 1 (4.8) | 15 (0.6) | -653.6% (-6602.8%,15.3%) | 0.07 | 0 (0.0) | 16 (0.7) | 100% | - |
| 2nd dose ≥14 days | 149 (4.9) | 116 (4.8) | 33 (5.0) | 17.1% (-24.1%,44.6%) | 0.363 | 1 (4.8) | 115 (4.8) | 34.6% (-400.4%,91.4%) | 0.683 | 0 (0.0) | 115 (4.8) | 100% | - |
| **Ad5-nCoV (CanSinoBIO)** |  |  |  |  |  |  |  |  |  |  |  |  |  |
| No vaccine | 2,899 (98.5) | 2,278 (98.4) | 621 (98.7) | Ref. |  | 19 (100.0) | 2,259 (98.4) | Ref. |  | 4 (100.0) | 2,247 (98.4) | Ref. |  |
| 1st dose ≥14 days | 32 (1.1) | 25 (1.1) | 7 (1.1) | -9.6 (-155.7%,53.1%) | 83% | 0 (0.0) | 25 (1.1) | 100% | - | 0 (0.0) | 25 (1.1) | 100% | - |
| 2nd dose ≥14 days | 13 (0.4) | 12 (0.5) | 1 (0.2) | -276.1% (-2815.3%,51.5%) | 0.205 | 0 (0.0) | 12 (0.5) | 100% | - | 0 (0.0) | 12 (0.5) | 100% | - |
| **mRNA-1273 (Moderna)** |  |  |  |  |  |  |  |  |  |  |  |  |  |
| No vaccine | 2,899 (91.1) | 2,278 (90.5) | 621 (93.5) | Ref. |  | 19 (95.0) | 2,259 (90.4) | Ref. |  | 4 (100.0) | 2,247 (90.3) | Ref. |  |
| 1st dose ≥14 days | 43 (1.4) | 38 (1.5) | 5 (0.8) | -132.1% (-493.5%,9.3%) | 0.079 | 0 (0.0) | 38 (1.5) | 100% | - | 0 (0.0) | 38 (1.5) | 100% | - |
| 2nd dose 0-13 days | 1 (0.0) | 1 (0.0) | 0 (0.0) | 0% |  | 0 (0.0) | 1 (0.0) | 100% | - | 0 (0.0) | 1 (0.04) | 100% | - |
| 2nd dose ≥14 days | 239 (7.5) | 201 (8.0) | 38 (5.7) | -72% (-147.9%,-19.3%) | 0.004 | 1 (5.0) | 200 (8.0) | -80% (-1396.4%,78.3%) | 0.586 | 0 (0.0) | 201 (8.1) | 100% | - |
| **Ad26.CoV2.S (Johnson & Johnson/Janssen)** |  |  |  |  |  |  |  |  |  |  |  |  |  |
| No vaccine | 2,899 (99.3) | 2,278 (99.2) | 621 (99.5) | Ref. |  | 19 (100.0) | 2,259 (99.2) | Ref. |  | 4 (100.0) | 2,247 (99.2) | Ref. |  |
| 1st dose ≥14 days | 17 (0.6) | 14 (0.6) | 3 (0.5) | -46.9% (-414.6%,58.1%) | 0.548 | 0 (0.0) | 14 (0.6) | 100% | - | 0 (0.0) | 14 (0.6) | 100% | - |
| 2nd dose ≥14 days | 4 (0.1) | 4 (0.2) | 0 (0.0) | 0% |  | 0 (0.0) | 4 (0.2) | 100% | - | 0 (0.0) | 4 (0.2) | 100% | - |
| **BBIBP-CorV (Sinopharm)** |  |  |  |  |  |  |  |  |  |  |  |  |  |
| No vaccine | 2,899 (99.9) | 2,278 (99.9) | 621 (98.8) | Ref. |  | 19 (100.0) | 2,259 (99.9) | Ref. |  | 4 (100.0) | 2,247 (99.9) | Ref. |  |
| 1st dose ≥14 days | 1 (0.0) | 0 (0.0) | 1 (0.29 | 100% |  | 0 (0.0) | 0 (0.0) | - | - | 0 (0.0) | 0 (0.0) | - | - |
| 2nd dose ≥14 days | 2 (0.1) | 2 (0.1) | 0 (0.0) | 0% |  | 0 (0.0) | 2 (0.1) | 100% | 100% | 0 (0.0) | 2 (0.1) | 100% | 100% |
| **NVX-CoV2373 (Novavax)** |  |  |  |  |  |  |  |  |  |  |  |  |  |
| No vaccine | 2,899 (100) | 2,278 (100.0) | 621 (100) | Ref. |  | 19 (100.0) | 2,259 (99.9) | Ref. |  | 4 (100.0) | 2,247 (99.9) | Ref. |  |
| 2nd dose ≥14 days | 1 (0.0) | 1 (0.0) | 0 (0.0) | 0% |  | 0 (0.0) | 1 (0.04) | 100% | 100% | 0 (0.0) | 1 (0.04) | 100% | 100% |

OR – Odd ratios, OR adjusted for sex, age, and tobacco smoking.
