# Supplementary material for: The impact of comorbidity status in COVID-19 vaccines effectiveness before and after SARS-CoV-2 omicron variant in northeastern Mexico: a retrospective multi-hospital study
Source: Front Public Health. 2024 Jun 12;12:1402527. doi: 10.3389/fpubh.2024.1402527 (PMC11199416; doi:10.3389/fpubh.2024.1402527)
Supplement: Supplementary file 1 [file Data_Sheet_1.ZIP › Table S2.docx]

**Table S2.** COVID-19 vaccines effectiveness in non-comorbid patients after Omicron.

| **Non-comorbid, after Omicron** | | | | | | | | | | | | | | | |
| --- | --- | --- | --- | --- | --- | --- | --- | --- | --- | --- | --- | --- | --- | --- | --- |
|  |  | COVID-19 infection | | | | Hospitalization | | | | Death | | | | | |
|  | Total | Yes | No | Effectiveness (95%CI) (Adjusted 1 – OR) | *p*-value | Yes | No | Effectiveness (95%CI) (Adjusted 1 – OR) | *p*-value | | Yes | No | Effectiveness (95%CI) (Adjusted 1 – OR) | *p*-value |  |
| **BNT162b2 (Pfizer)** |  |  |  |  |  |  |  |  |  | |  |  |  |  |  |
| No vaccine | 364,513 (96.0) | 117,769 (90.4) | 246,744 (98.9) | Ref. |  | 2,485 (94.8) | 115,284 (90.3) | Ref. |  | | 525 (97.6) | 115,093 (90.2) | Ref. |  |  |
| 1st dose 0-13 days | 29 (0.0) | 20 (0.0) | 9 (0.0) | -447.9% (-1105.2%,-149.1%) | <0.001 | 0 (0.0) | 20 (0.0) | 100% | 0.999 | | 0 (0.0) | 20 (0.0) | 100% | 0.999 |  |
| 1st dose ≥14 days | 1,325 (0.3) | 1,057 (0.8) | 268 (0.1) | -755.4% (-878.8%,-647.5%) | <0.001 | 11 (0.4) | 1,046 (0.8) | 33.2% (-25.1%,62.2%) | 0.22 | | 0 (0.0) | 1,053 (0.8) | 100% | 0.989 |  |
| 2nd dose 0-13 days | 96 (0.0) | 70 (0.1) | 26 (0.0) | -523.1% (-878.8%,-296.7%) | <0.001 | 0 (0.0) | 70 (0.1) | 100% | 0.997 | | 0 (0.0) | 70 (0.1) | 100% | 0.998 |  |
| 2nd dose ≥14 days | 13,885 (3.7) | 11,339 (8.7) | 2,546 (1.0) | -797.9% (-838.1%,-759.5%) | <0.001 | 124 (4.7) | 11,215 (8.8) | 44.5% (33.3%,53.7%) | <0.001 | | 13 (2.4) | 11,291 (8.9) | 62.2% (33.7%,78.4%) | 0.001 |  |
| **ChAdOx1 (AstraZeneca)** |  |  |  |  |  |  |  |  |  | |  |  |  |  |  |
| No vaccine | 364, 513 (96.1) | 117,769 (90.4) | 246,744 (99.1) | Ref. |  | 2,485 (97.1) | 115,284 (90.3) | Ref. |  | | 525(97.0) | 115,093 (90.2) | Ref. |  |  |
| 1st dose 0-13 days | 32 (0.0) | 28 (0.0) | 4 (0.0) | -1374.2% (-4112.7%,-415.9%) | <0.001 | 1 (0.0) | 27 (0.0) | -169.3% (-1907%,63.9%) | 0.334 | | 0 (0.0) | 28 (0.0) | 100% | 0.998 |  |
| 1st dose ≥14 days | 1,974 (0.5) | 1,609 (1.2) | 365 (0.1) | -805.7% (-915.5%,-707.8%) | <0.001 | 18 (0.7) | 1,591 (1.2) | 29.8% (-12.1%,56.1%) | 0.139 | | 3 (0.6) | 1,605 (1.3) | -24.5% (-298.7%,61.1%) | 0.712 |  |
| 2nd dose 0-13 days | 70 (0.0) | 59 (0.0) | 11 (0.0) | -943.9% (-1892.6%,-446.9%) | <0.001 | 0 (0.0) | 59 (0.0) | 100% | 0.997 | | 0 (0.0) | 59 (0.0) | 100% | 0.997 |  |
| 2nd dose ≥14 days | 12,764 (3.4) | 10,783 (8.3) | 1,981 (0.8) | -969.1% (-1022.3%,-918.4%) | <0.001 | 56 (2.2) | 10,727 (8.4) | 74.7% (66.9%,80.6%) | <0.001 | | 13 (2.4) | 10,742 (8.4) | 61.1% (31.8%,77.9%) | 0.001 |  |
| **CoronaVac (Sinovac)** |  |  |  |  |  |  |  |  |  | |  |  |  |  |  |
| No vaccine | 364,513 (99.2) | 117,769 (97.9) | 246,744 (99.8) | Ref. |  | 2,485 (99.2) | 115,284 (97.9) | Ref. |  | | 525 (99.1) | 115,093 (97.8) | Ref. |  |  |
| 1st dose 0-13 days | 4 (0.0) | 2 (0.0) | 1 (0.0) | -565.4% (-6348.8%,31.4%) | 0.102 | 0 (0.0) | 3 (0.0) | 100% | 0.999 | | 0 (0.0) | 3 (0.09 | 100% | - |  |
| 1st dose ≥14 days | 208 (0.1) | 176 (0.1) | 32 (0.0) | -915% (-1380.8%,-595.7%) | <0.001 | 0 (0.0) | 176 (0.1) | 100% | 0.995 | | 0 (0.0) | 176 (0.1) | 100% | - |  |
| 2nd dose 0-13 days | 6 (0.0) | 6 (0.0) | 0 (0.0) | 0.0% |  | 0 (0.0) | 6 (0.0) | 100% | 0.999 | | 0 (0.0) | 6 (0.09 | 100% | - |  |
| 2nd dose ≥14 days | 2,735 (0.7) | 2,357 (2.0) | 378 (0.2) | -1026.4% (-1156.4%,-909.9%) | <0.001 | 20 (0.8) | 2,337 (2.0) | 72.8% (57.7%,82.5%) | <0.001 | | 5 (0.9) | 2,350 (2.0) | 53% (-14.5%,80.7%) | 0.097 |  |
| **Ad5-nCoV (CanSinoBIO)** |  |  |  |  |  |  |  |  |  | |  |  |  |  |  |
| No vaccine | 364,513 (99.8) | 117,769 (99.6) | 246,744 (100.0) | Ref. |  | 2,485 (99.8) | 115,284 (99.6) | Ref. |  | | 525 (100.0) | 115,093 (99.6) | Ref. |  |  |
| 1st dose 0-13 days | 2 (0.0) | 2 (0.0) | 0 (0.0) | 0% |  | 0 (0.0) | 2 (0.0) | 100% |  | | 0 (0.0) | 2 (0.0) | 100% | - |  |
| 1st dose ≥14 days | 387 (0.1) | 312 (0.3) | 75 (0.0) | -745.5% (-989%,-556.4%) | <0.001 | 0 (0.0) | 312 (0.3) | 100% |  | | 0 (0.0) | 312 (0.3) | 100% | - |  |
| 2nd dose 0-13 days | 3 (0.0) | 2 (0.0) | 1 (0.0) | -334.9% (-4728.3%,60.8%) | 0.231 | 0 (0.0) | 2 (0.0) | 100% |  | | 0 (0.0) | 2 (0.0) | 100% | - |  |
| 2nd dose ≥14 days | 170 (0.0) | 143 (0.1) | 27 (0.0) | -942.9% (-1476%,-590.1%) | <0.001 | 4 (0.2) | 139 (0.1) | -44.8% (-294.5%,46.9%) | 0.47 | | 0 (0.0) | 142 (0.1) | 100% | - |  |
| **mRNA-1273 (Moderna)** |  |  |  |  |  |  |  |  |  | |  |  |  |  |  |
| No vaccine | 364,513 (98.6) | 117,769 (69.4) | 246,744 (99.6) | Ref. |  | 2,485 (99.1) | 115,284 (96.4) | Ref. |  | | 525 (100.0) | 115,093 (96.4) | Ref. |  |  |
| 1st dose 0-13 days | 7 (0.0) | 6 (0.0) | 1 (0.0) | -1172.3% (-10492.5%,-52.8%) | 0.019 | 0 (0.0) | 6 (0.0) | 100% | 0.999 | | 0 (0.0) | 6 (0.0) | 100% | - |  |
| 1st dose ≥14 days | 754 (0.2) | 607 (0.5) | 147 (0.1) | -775.8% (-950%,-630.6%) | <0.001 | 6 (0.2) | 601 (0.5) | 16.5% (-87.2%,62.8%) | 0.661 | | 0 (0.0) | 605 (0.5) | 100% | - |  |
| 2nd dose 0-13 days | 16 (0.0) | 11 (0.0) | 5 (0.0) | -359% (-1224.1%,-59.1%) | 0.005 | 0 (0.0) | 11 (0.0) | 100% | 0.999 | | 0 (0.0) | 11 (0.0) | 100% | - |  |
| 2nd dose ≥14 days | 4,534 (1.2) | 3,722 (3.0) | 812 (0.3) | -892.8% (-972%,-819.6%) | <0.001 | 16 (0.6) | 3,706 (3.1) | 59.4% (33.4%,75.3%) | <0.001 | | 0 (0.0) | 3,712 (3.1) | 100% | - |  |
| **Ad26.CoV2.S (Johnson & Johnson/Janssen)** |  |  |  |  |  |  |  |  |  | |  |  |  |  |  |
| No vaccine | 364,513 (99.9) | 117,769 (99.7) | 246,744 (100) | Ref. |  | 2,485 (100.0) | 115,284 (99.7) | Ref. |  | | 525 (100.0) | 115,093 (99.7) | Ref. |  |  |
| 1st dose 0-13 days | 1 (0.0) | 1 (0.0) | 0 (0.0) | 0.0% |  | 0 (0.0) | 1 (0.0) | 100% |  | | 0 (0.0) | 1 (0.0) | 100% | - |  |
| 1st dose ≥14 days | 295 (0.1) | 260 (0.2) | 35 (0.0) | -1543.7% (-2241.2%,-1054.1%) | <0.001 | 0 (0.0) | 260 (0.2) | 100% |  | | 0 (0.0) | 260 (0.2) | 100% | - |  |
| 2nd dose ≥14 days | 62 (0.0) | 49 (0.0) | 13 (0.0) | -709.4% (-1395.6%,-338%) | <0.001 | 0 (0.0) | 49 (0.04) | 100% | 1.000 | | 0 (0.0) | 48 (0.0) | 100% | - |  |
| **BBIBP-CorV (Sinopharm)** |  |  |  |  |  |  |  |  |  | |  |  |  |  |  |
| No vaccine | 364,531 (100) | 117,769 (100.0) | 246,744 (100.0) | Ref. |  | 2,485 (100.0) | 115,093 (100.0) | Ref. |  | | 525 (100.0) | 115,093 (100.0) | Ref. |  |  |
| 1st dose ≥14 days | 2 (0.0) | 0 (0.0) | 2 (0.0) | 100.0% | 0.999 | 0 (0.0) | 0 (0.0) | - |  | | - | - |  |  |  |
| 2nd dose ≥14 days | 24 (0.0) | 18 (0.0) | 6 (0.0) | -430.2% (-1247.9%,-108.5%) | <0.001 | 0 (0.0) | 18 (0.0) | 100% | 0.998 | | 0 (0.0) | 18 (0.0) | 100% | - |  |
| **NVX-CoV2373 (Novavax)** |  |  |  |  |  |  |  |  |  | |  |  |  |  |  |
| No vaccine | 364,513 (100.0) | 117,769 (100.0) | 246,744 (100.0) | Ref. |  | 2,485 (100.0) | 115,284 (100.0) | Ref. |  | | 525 (100.0) | 115,093 (100.0) | Ref. |  |  |
| 1st dose ≥14 days | 4 (0.0) | 4 (0.0) | 0 (0.0) | 0.0% | 0.999 | 0 (0.0) | 4 (0.0) | 100% |  | | 0 (0.0) | 4 (0.0) | 100% | - |  |
| 2nd dose 0-13 days | 1 (0.0) | 0 (0.0) | 1 (0.0) | 100.0% | 1.000 | 0 (0.0) | 0 (0.0) |  |  | | 0 (0.0) | 0 (0.0) | 100% | - |  |
| 2nd dose ≥14 days | 13 (0.0 | 12 (0.0) | 1 (0.0) | -2078.1% (-16679.1%,-182.7%) | 0.003 | 0 (0.0) | 12 (0.0) | 100% |  | | 0 (0.0) | 12 (0.0) | 100% | - |  |
| **Sputnik V (Gamaleya Institute)** |  |  |  |  |  |  |  |  |  | |  |  |  |  |  |
| No vaccine | 364,513 (100.0) | 117,769 (100.0) | 246,744 (100) | Ref. |  | 2,485 (100.0) | 115,284 (99.9) | Ref. |  | | 525 (100.0) | 115,093 (100.0) | Ref. |  |  |
| 1st dose ≥14 days | 11 (0.0) | 9 (0.0) | 2 (0.0) | -829.3% (-4246.2%,-98.7%) | 0.005 | 0 (0.0) | 9 (0.008) | 100% |  | | 0 (0.0) | 9 (0.0) | 100% | - |  |
| 2nd dose ≥14 days | 26 (0.0) | 21 (0.0) | 5 (0.0) | -809.2% (-2314.9%,-242.3%) | <0.001 | 0 (0.0) | 21 (0.02) | 100% |  | | 0 (0.0) | 21 (0.0) | 100% | - |  |

OR – Odd ratios, OR adjusted for sex, age and tobacco smoking.
