# Supplementary material for: The impact of comorbidity status in COVID-19 vaccines effectiveness before and after SARS-CoV-2 omicron variant in northeastern Mexico: a retrospective multi-hospital study
Source: Front Public Health. 2024 Jun 12;12:1402527. doi: 10.3389/fpubh.2024.1402527 (PMC11199416; doi:10.3389/fpubh.2024.1402527)
Supplement: Supplementary file 1 [file Data_Sheet_1.ZIP › Table S11.docx]

**Table S11.** COVID-19 vaccines effectiveness in patients with two comorbidities before Omicron.

| **Two comorbidities, before Omicron** | | | | | | | | | | | | | |
| --- | --- | --- | --- | --- | --- | --- | --- | --- | --- | --- | --- | --- | --- |
|  |  | COVID-19 infection | | | | Hospitalization | | | | Death | | | |
|  | Total | Yes | No | Effectiveness (95%CI) (Adjusted 1 – OR) | *p*-value | Yes | No | Effectiveness (95%CI) (Adjusted 1 – OR) | *p*-value | Yes | No | Effectiveness (95%CI) (Adjusted 1 – OR) | *p*-value |
| **BNT162b2 (Pfizer)** |  |  |  |  |  |  |  |  |  |  |  |  |  |
| No vaccine | 11,182 (92.9) | 3,941 (95.0) | 7,241 (91.7) | Ref. |  | 1,708 (97.4) | 2,233 (93.3) | Ref. |  | 977 (97.9) | 2,920 (94.0) | Ref. |  |
| 1st dose 0-13 days | 49 (0.4) | 15 (0.4) | 34 (0.4) | 12.6% (-61.2%,52.6%) | 0.667 | 0 (0.0) | 15 (0.6) | 100% (100%,100%) | 0.998 | 0 (0.0) | 15 (0.5) | 100% | - |
| 1st dose ≥14 days | 121 (1.0) | 34 (0.8) | 87 (1.1) | 26.1% (-10.2%,50.5%) | 0.137 | 10 (0.6) | 24 (1.0) | 43.7% (-23.2%,74.3%) | 0.151 | 5 (0.5) | 29 (0.9) | 41.8% (-55.8%,78.4%) | 0.280 |
| 2nd dose 0-13 days | 39 (0.3) | 7 (0.2) | 32 (0.4) | 59.9% (8.8%,82.3%) | 0.029 | 0 (0.0) | 7 (0.3) | 100% | - | 0 (0.0) | 7 (0.2) | 100% | - |
| 2nd dose ≥14 days | 650 (5.4) | 151 (3.6) | 499 (6.3) | 44.4% (32.9%,53.8%) | <0.001 | 36 (2.1) | 115 (4.8) | 70.9% (56.2%,80.6%) | <0.001 | 16 (1.6) | 134 (4.3) | 73.8% (54.6%,84.8%) | <0.001 |
| **ChAdOx1 (AstraZeneca)** |  |  |  |  |  |  |  |  |  |  |  |  |  |
| No vaccine | 11,182 (92.2) | 3,941 (91.9) | 7,241 (92.3) | Ref. |  | 1,708 (95.6) | 2,233 (89.2) | Ref. |  | 977 (95.7) | 2,920 (90.7) | Ref. |  |
| 1st dose 0-13 days | 86 (0.7) | 37 (0.9) | 49 (0.6) | -50.1% (-130.8%,2.4%) | 0.064 | 9 (0.5) | 28 (1.1) | 19.1% (-78.2%,63.2%) | 0.6 | 3 (0.3) | 34 (1.1) | 100% | - |
| 1st dose ≥14 days | 309 (2.5) | 130 (3.0) | 179 (2.3) | -42% (-78.8%,-12.8%) | 0.003 | 19 (1.1) | 111 (4.4) | 67.4% (45.4%,80.5%) | <0.001 | 12 (1.29 | 118 (3.7) | 49.1% (4.9%,72.7%) | 0.034 |
| 2nd dose 0-13 days | 40 (0.3) | 11 (0.3) | 29 (0.4) | 29.4% (-41.8%,34.8%) | 0.328 | 0 (0.0) | 11 (0.4) | 100% | - | 0 (0.0) | 11 (0.3) | 100% | - |
| 2nd dose ≥14 days | 515 (4.2) | 170 (4.0) | 345 (4.4) | 12.5% (-5.6%,27.6%) | 0.164 | 51 (2.9) | 119 (4.8) | 63.8% (48.2%,74.7%) | <0.001 | 29 (2.8) | 138 (4.3) | 58.3% (35.8%,72.9%) | <0.001 |
| **CoronaVac (Sinovac)** |  |  |  |  |  |  |  |  |  |  |  |  |  |
| No vaccine | 11,182 (96.6) | 3,941 (96.1) | 7,241 (96.9) | Ref. |  | 1,708 (98.3) | 2,233 (94.4) | Ref. |  | 977 (98.1) | 2,920 (95.4) | Ref. |  |
| 1st dose 0-13 days | 14 (0.1) | 6 (0.1) | 8 (0.1) | -36.4% (-294.3%,52.8%) | 0.566 | 3 (0.2) | 3 (0.1) | -65.7% (-752.7%,67.8%) | 0.546 | 2 (0.2) | 4 (0.1) | -130.2% (-1201.9%,59.3%) | 0.346 |
| 1st dose ≥14 days | 76 (0.7) | 27 (0.7) | 49 (0.7) | -3.4% (-65.8%,35.5%) | 0.89 | 7 (0.4) | 20 (0.8) | 36.4% (-53.1%,73.5%) | 0.313 | 5 (0.5) | 22 (0.7) | -13.9% (-0.6%,57.7%) | 0.796 |
| 2nd dose 0-13 days | 32 (0.3) | 20 (0.5) | 12 (0.2) | -222.3% (-560.9%,-57.2%) | 0.001 | 1 (0.1) | 19 (0.8) | 91.5% (35.9%,98.9%) | 0.017 | 1 (0.1) | 19 (0.6) | 76.8% (-75.3%,96.9%) | 0.157 |
| 2nd dose ≥14 days | 274 (2.4) | 109 (2.7) | 165 (2.2) | -22.5% (-56.6%,4.2%) | 0.105 | 19 (1.1) | 90 (3.8) | 71.6% (52.4%,82.9%) | <0.001 | 11 (1.1) | 97 (3.2) | 60.4% (24.7%,79.1%) | 0.005 |
| **Ad5-nCoV (CanSinoBIO)** |  |  |  |  |  |  |  |  |  |  |  |  |  |
| No vaccine | 11,182 (99.4) | 3,941 (99.2) | 7,241 (99.5) | Ref. |  | 1,708 (99.6) | 2,233 (98.9) | Ref. |  | 977 (99.7) | 2,920 (99.1) | Ref. |  |
| 1st dose 0-13 days | 3 (0.0) | 2 (0.1) | 1 (0.0) | -260% (-3896.8%,67.6%) | 0.297 | 1 (0.1) | 1 (0.0) | -24.8% (-2561.7%,94.2%) | 0.887 | 1 (0.1) | 1 (0.0) | -258.2% (-7987.8%,84.1%) | 0.422 |
| 1st dose ≥14 days | 58 (0.5) | 25 (0.6) | 33 (0.5) | -49.1% (-151.4%,11.6%) | 0.134 | 4 (0.2) | 21 (0.9) | 36.5% (-8.9%,87.8%) | 0.071 | 2 (0.2) | 23 (0.8) | 55.6% (-92%,89.7%) | 0.277 |
| 2nd dose ≥14 days | 6 (0.1) | 3 (0.1) | 3 (0.0) | -83.5% (-816.6%,63.3%) | 0.459 | 1 (0.1) | 2 (0.1) | 39.7% (-709.9%,95.5%) | 0.703 | 0 (0.0) | 3 (0.1) | 100% | - |
| **mRNA-1273 (Moderna)** |  |  |  |  |  |  |  |  |  |  |  |  |  |
| No vaccine | 11,182 (99.4) | 3,941 (99.6) | 7,241 (99.2) | Ref. |  | 1,708 (99.9) | 2,233 (99.5) | Ref. |  | 977 (99.9) | 2,920 (99.6) | Ref. |  |
| 1st dose 0-13 days | 8 (0.1) | 3 (0.1) | 5 (0.1) | -26.5% (-432.1%,69.9%) | 0.748 | 1 (0.1) | 2 (0.1) | -29.8% (-1553.5%,89.8%) | 0.841 | 0 (0.0) | 3 (0.1) | 100% | - |
| 1st dose ≥14 days | 23 (0.2) | 4 (0.1) | 19 (0.3) | 56% (-29.7%,85.1%) | 0.137 | 1 (0.1) | 3 (0.1) | 23.2% (-909.7%,94.2%) | 0.841 | 1 (0.1) | 3 (0.1) | -117.3% (-3012.4%,84.8%) | 0.568 |
| 2nd dose 0-13 days | 5 (0.0) | 0 (0.0) | 5 (0.1) | 100% | - | 0 (0.0) | 0 (0.0) | - | - |  |  |  |  |
| 2nd dose ≥14 days | 34 (0.3) | 7 (0.2) | 27 (0.4) | 46.5% (-23.2%,76.8%) | 0.142 | 0 (0.0) | 7 (0.3) | 100% | - | 0 (0.0) | 7 (0.2) | 100% | - |
| **Ad26.CoV2.S (Johnson & Johnson/Janssen)** |  |  |  |  |  |  |  |  |  |  |  |  |  |
| No vaccine | 11,182 (100.0) | 3,841 (100.0) | 7,241 (100.0) | Ref. |  | 1,708 (100.0) | 2,233 (100.0) | Ref. |  | 977 (100.0) | 2,920 (100.0) | Ref. |  |
| 1st dose ≥14 days | 2 (0.0) | 1 (0.0) | 1 (0.0) | -83.5% (-2843.7%,88.6%) | 0.668 | 0 (0.0) | 1 (0.0) | 100% | - | 0 (0.0) | 1 (0.0) | 100% | - |
| 2nd dose ≥14 days | 1 (0.0) | 0 (0.0) | 1 (0.0) | 100% | - | 0 (0.0) | 0 (0.0) | - | - | 0 (0.0) | 0 (0.0) | - | - |

OR – Odd ratios, OR adjusted for sex, age, and tobacco smoking.
