# Supplementary material for: The impact of comorbidity status in COVID-19 vaccines effectiveness before and after SARS-CoV-2 omicron variant in northeastern Mexico: a retrospective multi-hospital study
Source: Front Public Health. 2024 Jun 12;12:1402527. doi: 10.3389/fpubh.2024.1402527 (PMC11199416; doi:10.3389/fpubh.2024.1402527)
Supplement: Supplementary file 1 [file Data_Sheet_1.ZIP › Table S3.docx]

**Table S3.** COVID-19 vaccines effectiveness in patients with one comorbidity before Omicron.

| **With one comorbidity, before Omicron** | | | | | | | | | | | | | |
| --- | --- | --- | --- | --- | --- | --- | --- | --- | --- | --- | --- | --- | --- |
|  |  | COVID-19 infection | | | | Hospitalization | | | | Death | | | |
|  | Total | Yes | No | Effectiveness (95%CI) (Adjusted 1 – OR) | *p*-value | Yes | No | Effectiveness (95%CI) (Adjusted 1 – OR) | *p*-value | Yes | No | Effectiveness (95%CI) (Adjusted 1 – OR) | *p*-value |
| **BNT162b2 (Pfizer)** |  |  |  |  |  |  |  |  |  |  |  |  |  |
| No vaccine | 26,470 (93.3) | 9,538 (95.9) | 16,932 (91.9) | Ref. |  | 2,205 (97.9) | 7,333 (95.3) | Ref. |  | 1,125 (97.9) | 9,351 (95.6) | Ref. |  |
| 1st dose 0-13 days | 132 (0.5) | 48 (0.5) | 84 (0.5) | 2.2% (-39.8%,31.6%) | 0.901 | 9 (0.4) | 39 (0.5) | 23.7% (-68.1%,65.4%) | 0.501 | 6 (0.5) | 42 (0.5) | -16.4% (-200.7%,54.9%) | 0.754 |
| 1st dose ≥14 days | 273 (1.0) | 57 (0.6) | 216 (1.2) | 52.6% (36.4%,64.7%) | <0.001 | 8 (0.4) | 49 (0.6) | 52.7% (-5.9%,78.9%) | 0.069 | 4 (0.3) | 53 (0.6) | 48.7% (-55.4%,83.1%) | 0.238 |
| 2nd dose 0-13 days | 82 (0.3) | 16 (0.2) | 66 (0.4) | 55.7% (23.3%,74.4%) | 0.004 | 0 (0.0) | 16 (0.2) | 100% (100%,100%) | 0.995 | 0 (0.0) | 16 (0.2) | 100% (100%,100%) | 0.998 |
| 2nd dose ≥14 days | 1,407 (5.0) | 286 (2.9) | 1,121 (6.1) | 55.2% (48.8%,60.7%) | <0.001 | 31 (1.4) | 255 (3.3) | 71.8% (57.9%,81.1%) | <0.001 | 14 (1.2) | 270 (3.1) | 72.9% (52%,84.7%) | <0.001 |
| **ChAdOx1 (AstraZeneca)** |  |  |  |  |  |  |  |  |  |  |  |  |  |
| No vaccine | 26,470 (92.6) | 9,538 (92.0) | 9,538 (92.0) | Ref. |  | 2,205 (96.4) | 7,333 (90.7) | Ref. |  | 1,125 (97.2) | 8,351 (91.3) | Ref. |  |
| 1st dose 0-13 days | 282 (1.0) | 161 (1.6) | 161 (1.6) | -144.5% (-210.3%,-92.7%) | <0.001 | 15 (0.7) | 146 (1.8) | 54.1% (20.1%,73.6%) | 0.006 | 9 (0.8) | 151 (1.7) | 25.2% (-51.5%,63.1%) | 0.419 |
| 1st dose ≥14 days | 905 (3.2) | 405 (3.9) | 405 (3.9) | -48.4% (-69.7%,-29.8%) | <0.001 | 24 (1.0) | 381 (4.7) | 72.8% (58.2%,82.2%) | <0.001 | 7 (0.6) | 395 (4.3) | 78.4% (53.5%,90%) | <0.001 |
| 2nd dose 0-13 days | 119 (0.4) | 40 (0.4) | 40 (0.4) | 12.3% (-28.6%,40.3%) | 0.501 | 5 (0.2) | 35 (0.4) | 65.8% (10.3%,87%) | 0.029 | 1 (0.1) | 39 (0.4) | 85.1% (-11.1%,98%) | 0.063 |
| 2nd dose ≥14 days | 798 (2.8) | 225 (2.2) | 225 (2.2) | 36.8% (26.1%,46.1%) | <0.001 | 38 (1.7) | 187 (2.3) | 68.2% (53.6%,78.2%) | <0.001 | 16 (1.4) | 207 (2.3) | 74.8% (56.6%,85.3%) | <0.001 |
| **CoronaVac (Sinovac)** |  |  |  |  |  |  |  |  |  |  |  |  |  |
| No vaccine | 26,470 (97.4) | 9,538 (97.2) | 16,932 (97.5) | Ref. |  | 2,205 (97.9) | 7,333 (95.3) | Ref. |  | 1,125 (98.8) | 8,351 (96.9) | Ref. |  |
| 1st dose 0-13 days | 46 (0.2) | 21 (0.2) | 25 (0.1) | -51.3% (-171%,15.6%) | 0.164 | 9 (0.4) | 39 (0.5) | 23.7% (-68.1%,65.4%) | 0.501 | 1 (0.1) | 20 (0.2) | 47.7% (-305.7%,93.3%) | 0.535 |
| 1st dose ≥14 days | 171 (0.6) | 76 (0.8) | 95 (0.5) | -37.1% (-85.8%,-1.2%) | 0.042 | 8 (0.4) | 49 (0.6) | 52.7% (-5.9%,78.9%) | 0.069 | 4 (0.4) | 72 (0.8) | 62.6% (-0.6%,86.8%) | 0.066 |
| 2nd dose 0-13 days | 49 (0.2) | 30 (0.3) | 19 (0.1) | -164.1% (-370.3%,-48.3%) | 0.001 | 0 (0.0) | 16 (0.2) | 100% (100%,100%) | 0.998 | 1 (0.1) | 29 (0.3) | 75.5% (-81.9%,96.7%) | 0.169 |
| 2nd dose ≥14 days | 445 (1.6) | 152 (1.5) | 293 (1.7) | 15.7% (-2.9%,30.9%) | 0.092 | 31 (1.4) | 255 (3.3) | 71.8% (57.9%,81.1%) | <0.001 | 8 (0.7) | 143 (1.7) | 67.5% (32%,84.5%) | 0.003 |
| **Ad5-nCoV (CanSinoBIO)** |  |  |  |  |  |  |  |  |  |  |  |  |  |
| No vaccine | 26,470 (99.6) | 9,358 (99.6) | 16,932 (99.6) | Ref. |  | 2,205 (99.9) | 7,333 (99.5) | Ref. |  | 1,125 (99.9) | 8,351 (99.6) | Ref. |  |
| 1st dose 0-13 days | 5 (0.0) | 3 (0.0) | 2 (0.0) | -177.4% (-1606.3%,54.9%) | 0.271 | 1 (0.0) | 2 (0.0) | 13.2% (-1641.8%,95.7%) | 0.926 | 1 (0.1) | 2 (0.0) | -111.4% (-5439%,91.9%) | 0.653 |
| 1st dose ≥14 days | 100 (0.4) | 33 (0.3) | 67 (0.4) | 7.2% (-41.1%,39%) | 0.727 | 1 (0.0) | 32 (0.4) | 87.9% (10.6%,98.4%) | 0.038 | 0 (0.0) | 32 (0.4) | 100% | - |
| 2nd dose 0-13 days | 1 (0.0) | 0 (0.0) | 1 (0.0) | 100% | - | 0 (0.0) | 0 (0.0) | - | - | 0 (0.0) | 0 (0.0) | - | - |
| 2nd dose ≥14 days | 3 (0.0) | 1 (0.0) | 2 (0.0) | 26.2% (-735%,93.5%) | 0.806 | 0 (0.0) | 1 (0.0) | 100% | - | 0 (0.0) | 1 (0.0) | 100% | - |
| **mRNA-1273 (Moderna)** |  |  |  |  |  |  |  |  |  |  |  |  |  |
| No vaccine | 26,470 (98.7) | 9,538 (99.4) | 16,932 (98.3) | Ref. |  | 2,205 (99.9) | 7,333 (99.3) | Ref. |  | 1,125 (99.9) | 8,351 (99.4) | Ref. |  |
| 1st dose 0-13 days | 35 (0.1) | 12 (0.1) | 23 (0.1) | 1.9% (-97.8%,51.4%) | 0.957 | 1 (0.0) | 11 (0.19 | 40.3% (-391.1%,92.8%) | 0.631 | 1 (0.1) | 11 (0.1) | -111.4% (-5439%,91.9%) | 0.653 |
| 1st dose ≥14 days | 10 7(0.4) | 13 (0.1) | 94 (0.5) | 71.1% (48.4%,83.9%) | <0.001 | 0 (0.0) | 13 (0.2) | 100% | - | 0 (0.0) | 13 (0.2) | 100% | - |
| 2nd dose 0-13 days | 26 (0.1) | 1 (0.0) | 25 (0.1) | 92% (40.7%,98.9%) | 0.013 | 0 (0.0) | 1 (0.0) | 100% | - | 0 (0.0) | 1 (0.0) | 100% | - |
| 2nd dose ≥14 days | 173 (0.6) | 27 (0.3) | 146 (0.7) | 62.6% (43.5%,75.2%) | <0.001 | 1 (0.0) | 26 (0.4) | 62.4% (-195.1%,95.2%) | 0.352 | 0 (0.0) | 27 (0.3) | 100% | - |
| **Ad26.CoV2.S (Johnson & Johnson/Janssen)** |  |  |  |  |  |  |  |  |  |  |  |  |  |
| No vaccine | 26,470 (99.9) | 9,538 (99.9) | 16,932 (99.9) | Ref. |  | 2,205 (100.0) | 7,333 (99.9) | Ref. |  | 1,125 (100.0) | 8,351 (99.9) | Ref. |  |
| 1st dose 0-13 days | 2 (0.0) | 1 (0.0) | 1 (0.0) | -91.1% (-2957.1%,88.1%) | 0.647 | 0 (0.0) | 1 (0.0) | 100% | - | 0 (0.0) | 1 (0.0) | 100% | - |
| 1st dose ≥14 days | 14 (0.1) | 5 (0.1) | 9 (0.1) | -2.9% (-208.6%,65.7%) | 0.96 | 0 (0.0) | 5 (0.1) | 100% | - | 0 (0.0) | 5 (0.1) | 100% | - |
| 2nd dose ≥14 days | 2 (0.0) | 1 (0.0) | 1 (0.0) | -74.7% (-2697.1%,89.1%) | 0.693 | 0 (0.0) | 1 (0.0) | 100% | - | 0 (0.0) | 1 (0.0) | 100% | - |
| **BBIBP-CorV (Sinopharm)** |  |  |  |  |  |  |  |  |  |  |  |  |  |
| No vaccine | 26,470 (100.0) | 9,538 (100.0) | 16,932 (100.0) | Ref. |  | 2,205 (100.0) | 7,333 (100.0) | Ref. |  | 1,125 (100.0) | 8,351 (100.0) | Ref. |  |
| 1st dose ≥14 days | 1 (0.0) | 0 (0.0) | 1 (0.0) | 100% | - | 0 (0.0) | 0 (0.0) | - | - | 0 (0.0) | 0 (0.0) | - | - |
| 2nd dose ≥14 days | 2 (0.0) | 1 (0.0) | 1 (0.0) | -59.7% (-2472.5%,90.1%) | 0.741 | 0 (0.0) | 1 (0.0) | 100% | - | 0 (0.0) | 1 (0.0) | 100% | - |
| **NVX-CoV2373 (Novavax)** |  |  |  |  |  |  |  |  |  |  |  |  |  |
| No vaccine | 26,470 (100.0) | 9,538 (100.0) | 16,932 (100.0) | Ref. |  | 2,205 (100.0) | 7,333 (100.0) | Ref. |  | 1,125 (100.0) | 8,351 (100.0) | Ref. |  |
| 2nd dose ≥14 days | 4 (0.0) | 1 (0.0) | 3 (0.0) | 39.6% (-485.4%,93.8%) | 0.664 | 0 (0.0) | 1 (0.0) | 100% | - | 0 (0.0) | 1 (0.0) | 100% | - |

OR – Odd ratios, OR adjusted for sex, age, and tobacco smoking.
