# Supplementary material for: The impact of comorbidity status in COVID-19 vaccines effectiveness before and after SARS-CoV-2 omicron variant in northeastern Mexico: a retrospective multi-hospital study
Source: Front Public Health. 2024 Jun 12;12:1402527. doi: 10.3389/fpubh.2024.1402527 (PMC11199416; doi:10.3389/fpubh.2024.1402527)
Supplement: Supplementary file 1 [file Data_Sheet_1.ZIP › Table S12.docx]

**Table S12.** COVID-19 vaccines effectiveness in patients with two comorbidities before Omicron.

| **Two comorbidities, after Omicron** | | | | | | | | | | | | | |
| --- | --- | --- | --- | --- | --- | --- | --- | --- | --- | --- | --- | --- | --- |
|  |  | COVID-19 infection | | | | Hospitalization | | | | Death | | | |
|  | Total | Yes | No | Effectiveness (95%CI) (Adjusted 1 – OR) | *p*-value | Yes | No | Effectiveness (95%CI) (Adjusted 1 – OR) | *p*-value | Yes | No | Effectiveness (95%CI) (Adjusted 1 – OR) | *p*-value |
| **BNT162b2 (Pfizer)** |  |  |  |  |  |  |  |  |  |  |  |  |  |
| No vaccine | 5,926 (83.0) | 3,875 (80.2) | 2,051 (88.7) | Ref. |  | 508 (92.7) | 3,367 (78.6) | Ref. |  | 191 (90.5) | 3,613 (79.6) | Ref. |  |
| 1st dose 0-13 days | 4 (0.1) | 3 (0.1) | 1 (0.0) | -65.7% (-1496.5%,82.8%) | 0.662 | 1 (0.2) | 2 (0.0) | -366.3% (-6093.3%,64.9%) | 0.243 | 1 (0.5) | 2 (0.0) | -2042.3% (-35554.5%,-27.6%) | 0.033 |
| 1st dose ≥14 days | 63 (0.9) | 49 (1.0) | 14 (0.6) | -85.5% (-237.1%,-2.1%) | 0.04 | 3 (0.59 | 46 (1.1) | 51.8% (-60.6%,85.6%) | 0.234 | 1 (0.5) | 48 (1.1) | 51.6% (-274.8%,93.7%) | 0.487 |
| 2nd dose 0-13 days | 1 (0.0) | 0 (0.0) | 1 (0.0) | 100% | - | 0 (0.0) | 0 (0.0) | - | - | 0 (0.0) | 0 (0.0) | - | - |
| 2nd dose ≥14 days | 1,147 (16.1) | 902 (18.7) | 245 (10.6) | -92.5% (-124%,-65.5%) | <0.001 | 36 86.6) | 866 (20.2) | 69% (55.9%,78.2%) | <0.001 | 18 (8.5) | 877 (19.3) | 49.4% (16.2%,69.4%) | 0.008 |
| **ChAdOx1 (AstraZeneca)** |  |  |  |  |  |  |  |  |  |  |  |  |  |
| No vaccine | 5,926 (81.6) | 3,875 (78.8) | 2,051 (87.5) | Ref. |  | 508 (86.1) | 3,367 (77.8) | Ref. |  | 191 (88.0) | 3,613 (78.2) | Ref. |  |
| 1st dose 0-13 days | 3 (0.0) | 2 (0.0) | 1 (0.0) | 1.7% (-986.9%,91.1%) | 0.989 | 0 (0.0) | 2 (0.0) | 100% | - | 0 (0.0) | 2 (0.0) | 100% | - |
| 1st dose ≥14 days | 140 (1.9) | 99 (2.0) | 41 (1.7) | -25.8% (-82%,13%) | 0.223 | 7 (1.2) | 92 (2.1) | 38.9% (-37.3%,72.8%) | 0.233 | 1 (0.5) | 98 (2.1) | 75.3% (-86.2%,96.7%) | 0.175 |
| 2nd dose 0-13 days | 4 (0.1) | 0 (0.0) | 4 (0.2) | 100% | - | 0 (0.0) | 0 (0.0) | - | - | 0 (0.0) | 0 (0.0) | - | - |
| 2nd dose ≥14 days | 1,188 (16.4) | 942 (19.2) | 246 (10.5) | -100.5% (-133%,-72.5%) | <0.001 | 75 (12.7) | 867 (20.0) | 32.7% (12.3%,48.3%) | 0.003 | 25 (11.5) | 909 (19.7) | 32.6% (-4.9%,56.7%) | 0.08 |
| **CoronaVac (Sinovac)** |  |  |  |  |  |  |  |  |  |  |  |  |  |
| No vaccine | 5,926 (93.0) | 3,875 (91.3) | 2,051 (96.4) | Ref. |  | 508 (96.4) | 3,367 (90.6) | Ref. | p-value | 191 (96.0) | 3,613 (91.0) | Ref. |  |
| 1st dose ≥14 days | 30 (0.5) | 26 (0.6) | 4 (0.2) | -244.7% (-889.6%,-20%) | 0.021 | 0 (0.0) | 26 (0.7) | 100% | - | 0 (0.0) | 26 (0.7) | 100% | - |
| 2nd dose 0-13 days | 1 (0.0) | 1 (0.0) | 0 (0.0) | 0% | - | 0 (0.0) | 1 (0.0) | 100% | - | 0 (0.0) | 1 (0.0) | 100% | - |
| 2nd dose ≥14 days | 414 (6.5) | 341 (8.0) | 73 (3.4) | -144.2% (-216.2%,-88.5%) | <0.001 | 19 (3.6) | 322 (8.7) | 51.6% (21.7%,70.1%) | 0.003 | 8 (4.0) | 330 (8.3) | 24.4% (-58.4%,63.9%) | 0.458 |
| **Ad5-nCoV (CanSinoBIO)** |  |  |  |  |  |  |  |  |  |  |  |  |  |
| No vaccine | 5,926 (99.3) | 3,875 (99.1) | 2,051 (99.7) | Ref. |  | 508 (100.0) | 3,367 (99.0) | Ref. |  | 191 (100.0) | 3,613 (99.1) | Ref. |  |
| 1st dose ≥14 days | 18 (0.3) | 17 (0.4) | 1 (0.0) | -776.6% (-6496.8%,-16.5%) | 0.035 | 0 (0.0) | 17 (0.5) | 100% | - | 0 (0.0) | 17 (0.5) | 100% | - |
| 2nd dose ≥14 days | 22 (0.4) | 17 (0.4) | 5 (0.2) | -79.8% (-388.5%,33.8%) | 0.250 | 0 (0.0) | 17 (0.5) | 100% | - | 0 (0.0) | 17 (0.5) | 100% | - |
| **mRNA-1273 (Moderna)** |  |  |  |  |  |  |  |  |  |  |  |  |  |
| No vaccine | 5,926 (98.2) | 3,875 (98.0) | 2,051 (98.7) | Ref. |  | 508 (99.8) | 3,367 (97.7) | Ref. |  | 191 (100.0) | 3,613 (97.9) | Ref. |  |
| 1st dose ≥14 days | 21 (0.3) | 16 (0.4) | 5 (0.2) | -61.4% (-341.9%,41.1%) | 0.352 | 0 (0.0) | 16 (0.5) | 100% | - | 0 (0.0) | 16 (0.4) | 100% | - |
| 2nd dose ≥14 days | 85 (1.4) | 64 (1.6) | 21 (1.0) | -53.7% (-153.1%,6.7%) | 0.092 | 1 (0.2) | 63 (1.8) | 78.5% (-60.3%,97.1%) | 0.134 | 0 (0.0) | 63 (1.7) | 100% | - |
| **Ad26.CoV2.S (Johnson & Johnson/Janssen)** |  |  |  |  |  |  |  |  |  |  |  |  |  |
| No vaccine | 5,926 (99.9) | 3,875 (99.8) | 2,051 (100.0) | Ref. |  | 508 (100.0) | 3,367 (99.8) | Ref. |  | 191 (100.0) | 3,613 (99.8) | Ref. |  |
| 1st dose ≥14 days | 5 (0.1) | 5 (0.1) | 0 (0.0) | 0% | - | 0 (0.0) | 5 (0.1) | 100% | - | 0 (0.0) | 5 (0.1) | 100% | - |
| 2nd dose ≥14 days | 3 (0.1) | 3 (0.1) | 0 (0.0) | 0% | - | 0 (0.0) | 3 (0.1) | 100% | - | 0 (0.0) | 3 (0.1) | 100% | - |
| **BBIBP-CorV (Sinopharm)** |  |  |  |  |  |  |  |  |  |  |  |  |  |
| No vaccine | 5,926 (99.9) | 3,875 (99.8) | 2,051 (100.0) | Ref. |  | 508 (100.0) | 3,367 (99.8) | Ref. |  | 191 (100.0) | 3,613 (99.8) | Ref. |  |
| 2nd dose ≥14 days | 8 (0.1) | 7 (0.2) | 1 (0.0) | -278.1% (-2979.9%,54.6%) | 0.214 | 0 (0.09 | 7 (0.2) | 100% | - | 0 (0.0) | 7 (0.2) | 100% | - |
| **NVX-CoV2373 (Novavax)** |  |  |  |  |  |  |  |  |  |  |  |  |  |
| No vaccine | 5,926 (99.9) | 3,875 (99.9) | 2,051 (100.0) | Ref. |  | 508 (100.0) | 3,367 (99.9) | Ref. |  | 191 (100.0) | 3,613 (99.9) | Ref. |  |
| 1st dose ≥14 days | 1 (0.0) | 1 (0.0) | 0 (0.0) | 0% | - | 0 (0.0) | 1 (0.0) | 100% | - | 0 (0.0) | 1 (0.0) | 100% | - |
| 2nd dose ≥14 days | 2 (0.0) | 2 (0.1) | 0 (0.0) | 0% | - | 0 (0.0) | 2 (0.1) | 100% | - | 0 (0.0) | 2 (0.1) | 100% | - |

OR – Odd ratios, OR adjusted for sex, age, and tobacco smoking.
