# Supplementary material for: The impact of comorbidity status in COVID-19 vaccines effectiveness before and after SARS-CoV-2 omicron variant in northeastern Mexico: a retrospective multi-hospital study
Source: Front Public Health. 2024 Jun 12;12:1402527. doi: 10.3389/fpubh.2024.1402527 (PMC11199416; doi:10.3389/fpubh.2024.1402527)
Supplement: Supplementary file 1 [file Data_Sheet_1.ZIP › Table S9.docx]

**Table S9.** COVID-19 vaccines effectiveness in patients with Diabetes Mellitus before Omicron.

| **Diabetes Mellitus, before Omicron** | | | | | | | | | | | | | |
| --- | --- | --- | --- | --- | --- | --- | --- | --- | --- | --- | --- | --- | --- |
|  |  | COVID-19 infection | | | | Hospitalization | | | | Death | | | |
|  | Total | Yes | No | Effectiveness (95%CI) (Adjusted 1 – OR) | *p*-value | Yes | No | Effectiveness (95%CI) (Adjusted 1 – OR) | *p*-value | Yes | No | Effectiveness (95%CI) (Adjusted 1 – OR) | *p*-value |
| **BNT162b2 (Pfizer)** |  |  |  |  |  |  |  |  |  |  |  |  |  |
| No vaccine | 4,551 (94.5) | 1,812 (97.1) | 2,739 (92.8) | Ref. |  | 599 (98.5) | 1,213 (96.3) | Ref. |  | 308 (99.0) | 1,489 (96.6) | Ref. |  |
| 1st dose 0-13 days | 18 (0.4) | 7 (0.4) | 11 (0.4) | -0.5% (-160.9%,61.3%) | 0.973 | 2 (0.3) | 5 (0.4) | -17.6% (-611.2%,80.5%) | 0.860 | 1 (0.3) | 6 (0.4) | -17.8% (-1061.9%,88.1%) | 0.875 |
| 1st dose ≥14 days | 38 (0.8) | 11 (0.6) | 27 (0.9) | 35.6% (-30.5%,68.2%) | 0.222 | 2 (0.39 | 9 (0.7) | 53.1% (-141.5%,90.9%) | 0.365 | 1 (0.3) | 10 (0.6) | 44.2% (-373.8%,93.4%) | 0.596 |
| 2nd dose 0-13 days | 15 (0.3) | 3 (0.2) | 12 (0.4) | 61.8% (-36.2%,89.3%) | 0.138 | 0 (0.0) | 3 (0.2) | 100% | - | 0 (0.0) | 3 (0.2) | 100% | - |
| 2nd dose ≥14 days | 195 (4.0 | 34 (1.8) | 161 (5.5) | 67.5% (52.7%,77.7%) | <0.001 | 5 (0.8) | 29 (2.3) | 79.3% (43.3%,92.4%) | 0.002 | 1 (0.3) | 33 (2.1) | 90.7% (60.1%,98.8%) | 0.021 |
| **ChAdOx1 (AstraZeneca)** |  |  |  |  |  |  |  |  |  |  |  |  |  |
| No vaccine | 4,551 (92.2) | 1,812 (91.6) | 2,739 (92.7) | Ref. |  | 599 (96.5) | 1,213 (89.3) | Ref. |  | 308 (97.8) | 1,489 (90.4) | Ref. |  |
| 1st dose 0-13 days | 44 (0.9) | 24 (1.2) | 20 (0.7) | -91.7% (-249.3%,-10.52%) | 0.034 | 4 (0.6) | 20 (1.5) | 47.7% (-69%,83.8%) | 0.279 | 3 (1.0) | 21 (1.3) | 2.1% (-268.4%,74%) | 0.98 |
| 1st dose ≥14 days | 157 (3.2) | 79 (4.0) | 78 (2.6) | -66.3% (-129.1%,-20.6%) | 0.002 | 6 (1.0) | 73 (5.4) | 76.6% (43.4%,90.4%) | 0.001 | 0 (0.0) | 78 (4.7) | 100% | - |
| 2nd dose 0-13 days | 22 (0.4) | 10 (0.5) | 12 (0.4) | 69.3% (-204.2%,43.9%) | 0.535 | 1 (0.29 | 9 (0.7) | 80.6% (-57.9%,97.6%) | 0.125 | 0 (0.0) | 10 (0.6) | 100% | - |
| 2nd dose ≥14 days | 161 (3.3) | 54 (2.7) | 107 (3.6) | 27.9% (-0.9%,48.4%) | 0.055 | 11 (1.8) | 42 (3.2) | 69% (35.1%,85.2%) | 0.002 | 4 (1.3) | 50 (0.3) | 77.9% (34.2%,92.6%) | 0.007 |
| **CoronaVac (Sinovac)** |  |  |  |  |  |  |  |  |  |  |  |  |  |
| No vaccine | 4,551 (96.8) | 1,812 (96.7) | 2,739 (96.8) | Ref. |  | 599 (98.4) | 1,213 (96.0) | Ref. |  | 308 (99.4) | 1,489 (96.3) | Ref. |  |
| 1st dose 0-13 days | 8 (0.2) | 3 (0.2) | 5 (0.2) | 8.1% (-286.9%,78.2%) | 0.908 | 0 (0.0) | 3 (0.2) | 100% | - | 0 (0.0) | 3 (0.2) | 100% | - |
| 1st dose ≥14 days | 38 (0.8) | 17 (0.9) | 21 (0.7) | -29.8% (-147.4%,31.9%) | 0.429 | 6 (1.0) | 11 (0.9) | 1.8% (-179.7%,65.6%) | 0.972 | 2 (0.6) | 15 (1.0) | 40.1% (-0.6%,86.9%) | 0.57 |
| 2nd dose 0-13 days | 14 (0.3) | 10 (0.5) | 4 (0.1) | -284.2% (-1131.2%,-19.9%) | 0.024 | 0 (0.0) | 10 (0.8) | 100% | - | 0 (0.0) | 10 (0.6) | 100% | - |
| 2nd dose ≥14 days | 91 (1.9) | 31 (1.7) | 60 (2.1) | 24% (-17.9%,51.1%) | 0.22 | 4 (0.7) | 27 (2.1) | 70% (10.6%,90%) | 0.031 | 0 (0.0) | 30 (1.9) | 100% | - |
| **Ad5-nCoV (CanSinoBIO)** |  |  |  |  |  |  |  |  |  |  |  |  |  |
| No vaccine | 4,551 (99.6) | 1,812 (99.7) | 2,739 (99.6) | Ref. |  | 599 (100.0) | 1,213 (99.5) | Ref. |  | 308 (100.0) | 1,489 (99.6) | Ref. |  |
| 1st dose 0-13 days | 1 (0.0) | 0 (0.0) | 1 (0.0) | 0% | - | 0 (0.0) | 0 (0.0) | - | - | 0 (0.0) | 0 (0.0) | - | - |
| 1st dose ≥14 days | 16 (0.4) | 6 (0.3) | 10 (0.4) | -2% (-182.6%,63.2%) | 0.970 | 0 (0.0) | 6 (0.5) | 100% | - | 0 (0.0) | 6 (0.4) | 100% | 0.999 |
| 2nd dose ≥14 days | 1 (0.0) | 0 (0.09 | 1 (0.0) | 0% | - | 0 (0.0) | 0 (0.0) | - | - | 0 (0.0) | 0 (0.0) | - | - |
| **mRNA-1273 (Moderna)** |  |  |  |  |  |  |  |  |  |  |  |  |  |
| No vaccine | 4,551 (99.3) | 1,812 (99.7) | 2,739 (99.1) | Ref. |  | 599 (99.8) | 1,213 (99.6) | Ref. |  | 308 (99.7) | 1,489 (99.7) | Ref. |  |
| 1st dose 0-13 days | 3 (0.1) | 2 (0.1) | 1 (0.0) | -173.1% (-2924.9%,75.3%) | 0.413 | 1 (0.2) | 1 (0.1) | -78% (-2816.1%,89.1%) | 0.686 | 1 (0.3) | 1 (0.1) | -373.6% (-7682%,71.2%) | 0.268 |
| 1st dose ≥14 days | 13 (0.3) | 2 (0.1) | 11 (0.4) | 67.7% (-46.6%,92.9%) | 0.143 | 0 (0.0) | 2 (0.2) | 100% | - | 0 (0.0) | 2 (0.1) | 100% | - |
| 2nd dose 0-13 days | 3 (0.1) | 0 (0.0) | 3 (0.1) | 100% | - | 0 (0.0) | 0 (0.0) | - | - | 0 (0.0) | 0 (0.0) | - | - |
| 2nd dose ≥14 days | 12 (0.3) | 2 (0.1) | 10 (0.4) | 63.9% (-66.1%,92.2%) | 0.191 | 0 (0.0) | 2 (0.2) | 100% | - | 0 (0.0) | 2 (0.1) | 100% | - |
| **Ad26.CoV2.S (Johnson & Johnson/Janssen)** |  |  |  |  |  |  |  |  |  |  |  |  |  |
| No vaccine | 4,551 (99.9) | 1,812 (99.9) | 2,739 (99.9) | Ref. |  | 599 (100.0) | 1,213 (99.9) | Ref. |  | 308 (100.0) | 1,489 (99.9) | Ref. |  |
| 1st dose ≥14 days | 4 (0.1) | 1 (0.1) | 3 (0.1) | 40.5% (-477%,93.9%) | 0.654 | 0 (0.0) | 1 (0.1) | 100% | - | 0 (0.0) | 1 (0.1) | 100% | - |
| **NVX-CoV2373 (Novavax)** |  |  |  |  |  |  |  |  |  |  |  |  |  |
| No vaccine | 4,551 (100.0) | 1,812 (99.9) | 2,739 (100.0) | Ref. |  | 599 (100.0) | 1,213 (99.9) | Ref. |  | 308 (100.0) | 1,489 (99.9) | Ref. |  |
| 2nd dose ≥14 days | 2 (0.0) | 1 (0.1) | 1 (0.0) | -42.7% (-2186.5%,91.1%) | 0.802 | 0 (0.0) | 1 (0.1) | 100% | - | 0 (0.0) | 1 (0.1) | 100% | - |

OR – Odd ratios, OR adjusted for sex, age, and tobacco smoking.
