# Supplementary material for: The impact of comorbidity status in COVID-19 vaccines effectiveness before and after SARS-CoV-2 omicron variant in northeastern Mexico: a retrospective multi-hospital study
Source: Front Public Health. 2024 Jun 12;12:1402527. doi: 10.3389/fpubh.2024.1402527 (PMC11199416; doi:10.3389/fpubh.2024.1402527)
Supplement: Supplementary file 1 [file Data_Sheet_1.ZIP › Table S8.docx]

**Table S8.** COVID-19 vaccines effectiveness in patients with hypertension after Omicron.

| **Hypertension, after Omicron** | | | | | | | | | | | | |  |
| --- | --- | --- | --- | --- | --- | --- | --- | --- | --- | --- | --- | --- | --- |
|  |  | COVID-19 infection | | | | Hospitalization | | | | Death | | | |
|  | Total | Yes | No | Effectiveness (95%CI) (Adjusted 1 – OR) | *p*-value | Yes | No | Effectiveness (95%CI) (Adjusted 1 – OR) | *p*-value | Yes | No | Effectiveness (95%CI) (Adjusted 1 – OR) | *p*-value |
| **BNT162b2 (Pfizer)** |  |  |  |  |  |  |  |  |  |  |  |  |  |
| No vaccine | 4,287 (83.6) | 3,238 (81.9) | 1,049 (89.0) | Ref. |  | 162 (90.5) | 3,076 (81.5) | Ref. |  | 66 (94.3) | 3,144 (81.6) | Ref. |  |
| 1st dose ≥14 days | 41 (0.8) | 35 (0.9) | 6 (0.5) | -78.3% (-325.5%,25.3%) | 0.193 | 1 (0.6) | 34 (0.9) | 1.9% (-660.8%,87.3%) | 0.986 | 0 (0.0) | 35 (0.9) | 100% | - |
| 2nd dose 0-13 days | 4 (0.1) | 4 (0.1) | 0 (0.0) | 0% | - | 0 (0.0) | 4 (0.1) | 0% | - | 0 (0.0) | 4 (0.1) | 100% | - |
| 2nd dose ≥14 days | 799 (15.6) | 675 (17.1) | 124 (10.5) | -73.2% (-112.4%,-41.2%) | <0.001 | 16 (8.9) | 659 (17.5) | 47.6% (10.2%,69.4%) | 0.019 | 4 (5.7) | 670 (17.4) | 63.2% (-4.5%,87%) | 0.060 |
| **ChAdOx1 (AstraZeneca)** |  |  |  |  |  |  |  |  |  |  |  |  |  |
| No vaccine | 4,287 (81.2) | 3,238 (80.0) | 1,049 (52.1) | Ref. |  | 162 (81.8) | 3,076 (79.9) | Ref. |  | 66 (85.7) | 3,144 (79.8) | Ref. |  |
| 1st dose 0-13 days | 2 (0.0) | 1 (0.0) | 1 (0.1) | 74.4% (-309.9%,98.4%) | 0.335 | 0 (0.0) | 1 (0.0) | 100% | - | 0 (0.0) | 1 (0.0) | 100% | 1.000 |
| 1st dose ≥14 days | 108 (2.0) | 83 (2.0) | 25 (2.0) | 0.6% (-56.7%,36.9%) | 0.98 | 5 (2.5) | 78 (2.0) | 0% | - | 2 (2.6) | 81 (2.1) | -250% (-1709.2%,32.3%) | 0.135 |
| 2nd dose 0-13 days | 5 (0.1) | 4 (0.1) | 1 (0.1) | -3.7% (-832.9%,98.85%) | 0.989 | 0 (0.0) | 4 (0.1) | 100% | - | 0 (0.0) | 4 (0.1) | 100% | - |
| 2nd dose ≥14 days | 879 (16.6) | 723 (17.9) | 156 (12.7) | -47.2% (-77.5%,-22%) | <0.001 | 31 (15.7) | 692 (18.0) | 3.6% (-47%,36.8%) | 0.864 | 9 (11.7) | 710 (18.0) | 33% (-42%,68.4%) | 0.296 |
| **CoronaVac (Sinovac)** |  |  |  |  |  |  |  |  |  |  |  |  |  |
| No vaccine | 4,287 (91.1) | 3,238(89.9) | 1,049 (94.9) | Ref. |  | 162 (97.6) | 3,076 (89.5) | Ref. |  | 66 (100.0) | 3,144 (89.6) | Ref. |  |
| 1st dose ≥14 days | 37 (0.8) | 35 (1.0) | 2 (0.2) | -457.9% (-2226.3%,-33.8%) | 0.018 | 1 (0.6) | 34 (1.0) | 13.2% (-457.8%,91.6%) | 0.725 | 0 (0.0) | 35 (1.0) | 100% | - |
| 2nd dose 0-13 days | 1 (0.0) | 1 (0.0) | 0 (0.0) | 0% | - | 0 (0.0) | 1 (0.0) | 100% | - | 0 (0.0) | 1 (0.0) | 100% | - |
| 2nd dose ≥14 days | 383 (8.1) | 329 (9.1) | 54 (4.9) | -92% (-158.4%,-42.7%) | <0.001 | 3 (1.8) | 326 (9.5) | 70.5% (5.5%,90.8%) | 0.039 | 0 (0.0) | 328 (9.4) | 100% | - |
| **Ad5-nCoV (CanSinoBIO)** |  |  |  |  |  |  |  |  |  |  |  |  |  |
| No vaccine | 4,287 (99.3) | 3,238 (99.2) | 1,049 (99.7) | Ref. |  | 162 (99.4) | 3,076 (99.2) | Ref. |  | 66 (100.0) | 3,144 (99.1) | Ref. |  |
| 1st dose ≥14 days | 21 (0.5) | 18 (0.6) | 3 (0.3) | -75.5% (-497.9%,48.5%) | 0.369 | 0 (0.0) | 18 (0.6) | 100% | - | 0 (0.0) | 18 (0.6) | 100% | - |
| 2nd dose 0-13 days | 1 (0.0) | 1 (0.0) | 0 (0.0) | 0% | 1.000 | 0 (0.0) | 1 (0.0) | 100% | - | 0 (0.0) | 1 (0.0) | 100% | - |
| 2nd dose ≥14 days | 8 (0.2) | 8 (0.2) | 0 (0.0) | 0% | 0.999 | 1 (0.6) | 7 (0.2) | -390.3% (-4241.4%,44.6%) | 0.153 | 0 (0.0) | 8 (0.3) | 100% | - |
| **mRNA-1273 (Moderna)** |  |  |  |  |  |  |  |  |  |  |  |  |  |
| No vaccine | 4,287 (98.0) | 3,238 (97.8) | 1,049 (98.6) | Ref. |  | 162 (98.8) | 3,076 (97.7) | Ref. |  | 66 (98.5) | 3,144 (97.8) | Ref. |  |
| 1st dose ≥14 days | 14 (0.3) | 13 (0.4) | 1 (0.1) | -310.3% (-3063.7%,46.8%) | 0.176 | 0 (0.0) | 13 (0.4) | 100% | - | 0 (0.0) | 13 (0.4) | 100% | - |
| 2nd dose ≥14 days | 74 (1.7) | 60 (1.8) | 14 (1.3) | -22.1% (-120.4%,32.3%) | 0.507 | 2 (1.2) | 58 (1.8) | -75.3% (-697.9%,61.5%) | 0.468 | 1 (1.5) | 59 (1.8) | -260.3% (-3759.3%,66.4%) | 0.289 |
| **Ad26.CoV2.S (Johnson & Johnson/Janssen)** |  |  |  |  |  |  |  |  |  |  |  |  |  |
| No vaccine | 4,287 (99.9) | 3,238 (99.9) | 1,049 (99.7) | Ref. |  | 162 (100.0) | 3,076 (99.9) | Ref. |  | 66 (100.0) | 3,144 (99.9) | Ref. |  |
| 1st dose ≥14 days | 4 (0.1) | 1 (0.0) | 3 (0.3) | 91.7% (20%,90.1%) | 0.031 | 0 (0.0) | 1 (0.0) | 100% | - | 0 (0.0) | 1 (0.0) | 100% | - |
| 2nd dose ≥14 days | 1 (0.0) | 1 (0.0) | 0 (0.0) | 0% | - | 0 (0.0) | 1 (0.0) | 100% | - | 0 (0.0) | 1 (0.0) | 100% | - |
| **BBIBP-CorV (Sinopharm)** |  |  |  |  |  |  |  |  |  |  |  |  |  |
| No vaccine | 4,287 (99.9) | 3,238 (99.9) | 1,049 (100.0) | Ref. |  | 162 (99.4) | 3,076 (99.9) | Ref. |  | 66 (100.0) | 3,144 (99.9) | Ref. |  |
| 2nd dose ≥14 days | 3 (0.1) | 3 (0.1) | 0 (0.0) | 0% | - | 1 (0.6) | 2 (0.1) | -639.6% (-9663.2%,44%) | 0.129 | 0 (0.0) | 3 (0.1) | 100% | - |
| **NVX-CoV2373 (Novavax)** |  |  |  |  |  |  |  |  |  |  |  |  |  |
| No vaccine | 4,287 (99.9) | 3,238 (99.9) | 1,049 (100.0) | Ref. |  | 162 (99.4) | 3,076 (99.9) | Ref. |  | 66 (100.0) | 3,144 (99.9) | Ref. |  |
| 2nd dose ≥14 days | 3 (0.1) | 3 (0.1) | 0 (0.0) | 100% | - | 1 (0.6) | 2 (0.1) | -1486.1% (-20672.8%,-21.1%) | 0.035 | 0 (0.0) | 3 (0.1) | 100% | - |

OR – Odd ratios, OR adjusted for sex, age, and tobacco smoking.
