# Supplementary material for: The impact of comorbidity status in COVID-19 vaccines effectiveness before and after SARS-CoV-2 omicron variant in northeastern Mexico: a retrospective multi-hospital study
Source: Front Public Health. 2024 Jun 12;12:1402527. doi: 10.3389/fpubh.2024.1402527 (PMC11199416; doi:10.3389/fpubh.2024.1402527)
Supplement: Supplementary file 1 [file Data_Sheet_1.ZIP › Table S7.docx]

**Table S7.** COVID-19 vaccines effectiveness in patients with hypertension before Omicron.

| **Hypertension, before Omicron** | | | | | | | | | | | | | |
| --- | --- | --- | --- | --- | --- | --- | --- | --- | --- | --- | --- | --- | --- |
|  |  | COVID-19 infection | | | | Hospitalization | | | | Death | | | |
|  | Total | Yes | No | Effectiveness (95%CI) (Adjusted 1 – OR) | *p*-value | Yes | No | Effectiveness (95%CI) (Adjusted 1 – OR) | *p*-value | Yes | No | Effectiveness (95%CI) (Adjusted 1 – OR) | *p*-value |
| **BNT162b2 (Pfizer)** |  |  |  |  |  |  |  |  |  |  |  |  |  |
| No vaccine | 6,309 (92.5) | 2,457 (95.2) | 3,852 (90.9) | Ref. |  | 746 (97.5) | 1,711 (94.2) | Ref. |  | 577 (96.2) | 5,702 (92.1) | Ref. |  |
| 1st dose 0-13 days | 33 (0.5) | 9 (0.3) | 24 (0.6) | 44.5% (-19.9%,74.3%) | 0.134 | 4 (0.5) | 5 (0.3) | -31.6% (-480.6%,70.2%) | 0.717 | 5 (0.8) | 28 (0.5) | -236.2% (-1418.4%,25.5%) | 0.115 |
| 1st dose ≥14 days | 61 (0.9) | 14 (0.5) | 47 (1.1) | 51.7% (11.8%,73.6%) | 0.018 | 2 (0.3) | 12 (0.7) | 69.9% (-56%,94.2%) | 0.153 | 4 (0.7) | 57 (0.9) | 14% (-357.4%,83.8%) | 0.859 |
| 2nd dose 0-13 days | 15 (0.2) | 6 (0.2) | 9 (0.2) | -4.9% (-196.5%,62.9%) | 0.928 | 0 (0.0) | 6 (0.3) | 100% | - | 0 (0.0) | 15 (0.2) | 100% | - |
| 2nd dose ≥14 days | 401 (5.9) | 96 (3.7) | 305 (7.3) | 50.5% (37.3%,60.9%) | <0.001 | 13 (1.7) | 83 (4.6) | 69.3% (41.3%,84%) | <0.001 | 14 (2.3) | 386 (6.2) | 64.8% (18.2%,84.9%) | 0.015 |
| **ChAdOx1 (AstraZeneca)** |  |  |  |  |  |  |  |  |  |  |  |  |  |
| No vaccine | 6,309 (92.2) | 2,457 (91.8) | 3,852 (92.4) | Ref. |  | 746 (97.0) | 1,711 (89.7) | Ref. |  | 577 (97.0) | 5,702 (91.7) | Ref. |  |
| 1st dose 0-13 days | 73 (1.1) | 45 (1.7) | 28 (0.7) | -167.6% (-331%,-66.2%) | <0.001 | 2 (0.3) | 43 (2.3) | 83.5% (29.7%,96.1%) | 0.015 | 3 (0.5) | 70 (1.1) | 53.6% (-99.7%,89.2%) | 0.302 |
| 1st dose ≥14 days | 173 (2.5) | 79 (3.0) | 94 (2.3) | -36.2% (-84.8%,-0.4%) | 0.047 | 6 (0.8) | 73 (3.8) | 69% (24.4%,87.3%) | 0.01 | 5 (0.8) | 168 (2.7) | 61.7% (-31.2%,88.8%) | 0.126 |
| 2nd dose 0-13 days | 31 (0.5) | 15 (0.6) | 16 (0.4) | -41.2% (-186.9%,30.5%) | 0.34 | 3 (0.4) | 12 (0.6) | 35.7% (-148.1%,83.3%) | 0.522 | 1 (0.2) | 30 (0.5) | 59.5% (-240.5%,95.2%) | 0.405 |
| 2nd dose ≥14 days | 259 (3.8) | 80 (3.0) | 179 (4.3) | 30.9% (9.5%,47.3%) | 0.007 | 12 (1.6) | 68 (3.6) | 79.4% (60%,89.4%) | <0.001 | 9 (1.5) | 247 (4.0) | 76.8% (44.1%,90.4%) | 0.001 |
| **CoronaVac (Sinovac)** |  |  |  |  |  |  |  |  |  |  |  |  |  |
| No vaccine | 6,309 (96.1) | 2,457 (96.1) | 3,852 (96.1) | Ref. |  | 746 (98.9) | 1,711 (94.9) | Ref. |  | 577 (98.1) | 5,702 (95.9) | Ref. |  |
| 1st dose 0-13 days | 12 (0.2) | 6 (0.2) | 6 (0.1) | -57.9% (-392.4%,49.3%) | 0.431 | 0 (0.0) | 6 (0.3) | 100% | - | 0 (0.0) | 12 (0.2) | 100% | - |
| 1st dose ≥14 days | 56 (0.9) | 25 (1.0) | 31 (0.8) | -33% (-126.5%,21.9%) | 0.293 | 1 (0.1) | 24 (1.3) | 84.4% (-17.7%,97.9%) | 0.072 | 1 (0.2) | 55 (0.9) | 100% (-0.6%,100%) | 0.998 |
| 2nd dose 0-13 days | 12 (0.2) | 7 (0.3) | 5 8(0.1) | -115.3% (-582.2%,32%) | 0.192 | 0 (0.0) | 7 (0.4) | 100% | - | 0 (0.0) | 12 (0.2) | 100% | - |
| 2nd dose ≥14 days | 177 (2.7) | 62 (2.4) | 115 (2.9) | 15.4% (-15.9%,38.2%) | 0.298 | 7 (0.9) | 55 (3.1) | 97.28% (37%,88.3%) | 0.002 | 10 (1.7) | 167 (2.8) | 40.9% (-47.6%,76.3%) | 0.26 |
| **Ad5-nCoV (CanSinoBIO)** |  |  |  |  |  |  |  |  |  |  |  |  |  |
| No vaccine | 6,309 (99.7) | 2,457 (99.6) | 3,852 (99.7) | Ref. |  | 746 (99.9) | 1,711 (99.5) | Ref. |  | 577 (99.8) | 5,702 (99.6) | Ref. |  |
| 1st dose 0-13 days | 1 (0.0) | 1 (0.0) | 0 (0.0) | 100% (100%,100%) | 1.000 | 1 (0.1) | 0 (0.0) | 0% | - | 1 (0.2) | 0 (0.0) | 0% | - |
| 1st dose ≥14 days | 19 (0.3) | 9 (0.4) | 10 (0.3) | -39.9% (-246.1%,43.4%) | 0.467 | 0 (0.0) | 9 (0.5) | 100% | - | 0 (0.0) | 19 (0.3) | 100% | - |
| 2nd dose 0-13 days | 1 (0.0) | 0 (0.0) | 1 (0.0) | 100% | - | 0 (0.0) | 0 (0.0) | - | - | 0 (0.0) | 1 (0.0) | - |  |
| 2nd dose ≥14 days | 1 (0.0) | 0 (0.0) | 1 (0.0) | 100% | - | 0 (0.0) | 0 (0.0) | - | - | 0 (0.0) | 1 (0.0) | - | - |
| **mRNA-1273 (Moderna)** |  |  |  |  |  |  |  |  |  |  |  |  |  |
| No vaccine | 6,309 (99.4) | 2,457 (99.8) | 3,852 (99.2) | Ref. |  | 746 (100.0) | 1,711 (99.8) | Ref. |  | 577 (99.4) | 5,702 (99.4) | Ref. |  |
| 1st dose 0-13 days | 6 (0.1) | 1 (0.0) | 5 (0.1) | 69.1% (-165.8%,96.4%) | 0.285 | 0 (0.0) | 1 (0.1) | 100% | - | 0 (0.0) | 6 (0.1) | 100% | - |
| 1st dose ≥14 days | 9 (0.1) | 1 (0.02) | 8 (0.2) | 76.6% (-87.9%,97.1%) | 0.172 | 0 (0.0) | 1 (0.1) | 100% | - | 0 (0.0) | 9 (0.2) | 100% | - |
| 2nd dose 0-13 days | 2 (0.0) | 0 (0.0) | 2 (0.1) | 100% | - | 0 (0.0) | 0 (0.0) | - | - | 0 (0.0) | 2 (0.0) | 100% | - |
| 2nd dose ≥14 days | 19 (0.3) | 2 (0.1) | 17 (0.4) | 80.8% (16.6%,95.6%) | 0.028 | 0 (0.0) | 2 (0.1) | 100% | - | 0 (0.0) | 19 (0.3) | 100% | - |

OR – Odd ratios, OR adjusted for sex, age, and tobacco smoking.
