# Supplementary material for: The impact of comorbidity status in COVID-19 vaccines effectiveness before and after SARS-CoV-2 omicron variant in northeastern Mexico: a retrospective multi-hospital study
Source: Front Public Health. 2024 Jun 12;12:1402527. doi: 10.3389/fpubh.2024.1402527 (PMC11199416; doi:10.3389/fpubh.2024.1402527)
Supplement: Supplementary file 1 [file Data_Sheet_1.ZIP › Table S1.docx]

| **Non-comorbid, before Omicron** | | | | | | | | | | | | | |
| --- | --- | --- | --- | --- | --- | --- | --- | --- | --- | --- | --- | --- | --- |
|  |  | COVID-19 infection | | | | Hospitalization | | | | Death | | | |
|  | Total | Yes | No | Effectiveness (95%CI) (Adjusted 1 – OR) | *p*-value | 00Yes | No | Effectiveness (95%CI) (Adjusted 1 – OR) | *p*-value | Yes | No | Effectiveness (95%CI) (Adjusted 1 – OR) | *p*-value |
| **BNT162b2 (Pfizer)** |  |  |  |  |  |  |  |  |  |  |  |  |  |
| No vaccine | 161,802 (96.4) | 57,310 (97.7) | 104,492 (95.7) | Ref. |  | 5,054 (98.4) | 52,256 (97.6) | Ref. |  | 1,924 (99.0) | 55,228 (97.7) | Ref. |  |
| 1st dose 0-13 days | 486 (0.3) | 201 (0.3) | 285 (0.3) | -30.6% (-56.6%,-989%) | 0.005 | 7 (0.1) | 194 (0.4) | 52.8% (-2.5%,78.3%) | <0.001 | 1 (0.1) | 200 (0.4) | 77.3% (-70%,97%) | 0.149 |
| 1st dose ≥14 days | 1,110 (0.7) | 288 (0.5) | 822 (0.8) | 35% (25.6%,43.2%) | <0.001 | 14 (0.3) | 274 (0.5) | 47.4% (-2.5%,70%) | <0.001 | 5 (0.3) | 282 (0.5) | 41.7% (49.1%,77.5%) | 0.266 |
| 2nd dose 0-13 days | 284 (0.2) | 39 (0.1) | 245 (0.2) | 70.2% (58.2%,78.8%) | <0.001 | 2 (0.0) | 37 (0.1) | 52% (-109%,89%) | <0.001 | 0 (0.0) | 39 (0.1) | 100% | - |
| 2nd dose ≥14 days | 4,138 (2.5) | 812 (1.4) | 3,326 (3.0) | 55.3% (51.7%,58.6%) | <0.001 | 57 (1.1) | 755 (1.4) | 44.5% (26%,58.4%) | <0.001 | 13 (0.7) | 795 (1.4) | 71.9% (49.4%,84.4%) | <0.001 |
| **ChAdOx1 (AstraZeneca)** |  |  |  |  |  |  |  |  |  |  |  |  |  |
| No vaccine | 161,802 (96.2) | 57,310 (95.8) | 104,492 (96.5) | Ref. |  | 5,054 (98.3) | 52,256 (95.5) | Ref. |  | 1,924 (98.8) | 55,228 (95.7) | Ref. |  |
| 1st dose 0-13 days | 1,094 (0.7) | 588 (1.0) | 506 (0.5) | -113% (-140%,-88.9%) | 0.005 | 16 (0.3) | 572 (1.0) | 64.3% (41%,78.4%) | <0.001 | 3 (0.2) | 583 (1.0) | 69.5% (4.7%,90.3%) | 0.041 |
| 1st dose ≥14 days | 3,082 (1.8) | 1,369 (2.3) | 1,713 (1.6) | -46% (-56.9%,-35.9%) | <0.001 | 33 (0.6) | 1,336 (2.4) | 74.2% (63.3%,81.8%) | <0.001 | 5 (0.3) | 1,362 (2.4) | 85.2% (63.9%,93.9%) | <0.001 |
| 2nd dose 0-13 days | 326 (0.2) | 84 (0.1) | 242 (0.2) | 36.3% (18.3%,50.3%) | <0.001 | 3 (0.1) | 81 (0.1) | 69.4% (3.3%,90.4%) | <0.001 | 0 (0.0) | 84 (0.1) | 100% | - |
| 2nd dose ≥14 days | 1,875 (1.1) | 493 (0.8) | 1,382 (1.3) | 37% (30.1%,43.2%) | <0.001 | 35 (0.7) | 458 (0.8) | 66.2% (51.1%,76.7%) | <0.001 | 16 (0.8) | 475 (0.8) | 70.3% (48.2%,83%) | <0.001 |
| **CoronaVac (Sinovac)** |  |  |  |  |  |  |  |  |  |  |  |  |  |
| No vaccine | 161,802 (98.9) | 57,310 (98.6) | 104,492 (99.1) | Ref. |  | 5,054 (99.2) | 52,256 (98.5) | Ref. |  | 1,924 (99.2) | 55,228 (98.6) | Ref. |  |
| 1st dose 0-13 days | 124 (0.1) | 69 (0.1) | 55 (0.1) | -132.8% (-232.2%,-63.1%) | <0.001 | 1 (0.0) | 68 (0.1) | 80.6% (-41.2%,97.3%) | 0.004 | 1 (0.1) | 68 (0.1) | 13.6% (-537%,88.3%) | 0.886 |
| 1st dose ≥14 days | 417 (0.3) | 209 (0.4) | 208 (0.2) | -77% (-114.7%,-45.9%) | <0.001 | 11 (0.2) | 198 (0.4) | 63.6% (32.4%,80.4%) | <0.001 | 3 (0.2) | 205 (0.4) | 68.6% (-0.6%,90.2%) | 0.052 |
| 2nd dose 0-13 days | 141 (0.1) | 79 (0.1) | 62 (0.1) | -126.2% (-215.9%,-62%) | <0.001 | 4 (0.1) | 75 (0.1) | 65.3% (4.3%,87.4%) | 0.001 | 1 (0.1) | 78 (0.1) | 70.3% (-115.3%,95.9%) | 0.23 |
| 2nd dose ≥14 days | 1,065 (0.7) | 469 (0.8) | 596 (0.6) | -35.8% (-53.4%,-20.2%) | 0.001 | 23 (0.5) | 446 (0.8) | 75.3% (62.1%,83.9%) | <0.001 | 10 (0.5) | 458 (0.8) | 70.2% (43%,84.5%) | <0.001 |
| **Ad5-nCoV (CanSinoBIO)** |  |  |  |  |  |  |  |  |  |  |  |  |  |
| No vaccine | 161,802 (99.7) | 57,310 (99.8) | 104,492 (99.7) | Ref. |  | 5,054 (99.9) | 52,256 (99.8) | Ref. |  | 1,924 (99.9) | 55,228 (99.8) | Ref. |  |
| 1st dose 0-13 days | 23 (0.0) | 5 (0.0) | 18 (0.0) | 44.9% (-48.6%,79.6%) | 0.227 | 0 (0.0) | 5 (0.0) | 100% | - | 0 (0.0) | 5 (0.0) | 100% | - |
| 1st dose ≥14 days | 409 (0.3) | 117 (0.2) | 292 (0.3) | 22% (3.3%,36.71%) | 0.023 | 3 (0.1) | 114 (0.2) | 72.5% (11.8%,91.4%) | <0.001 | 1 (0.1) | 116 (0.2) | 66.5% (-148.7%,94.6%) | 0.285 |
| 2nd dose 0-13 days | 5 (0.0) | 2 (0.0) | 3 (0.0) | -34.5% (-705.6%,77.5%) | 0.743 | 0 (0.0) | 2 (0.0) | 100% | - | 0 (0.0) | 2 (0.0) | 100% | - |
| 2nd dose ≥14 days | 14 (0.0) | 2 (0.0) | 12 (0.0) | 71.1% (-29.3%,93.6%) | 0.106 | 1 (0.0) | 1 (0.0) | -751.7% (-14804.9%,51.3%) | 0.472 | 0 (0.0) | 2 (0.0) | 100% | - |
| **mRNA-1273 (Moderna)** |  |  |  |  |  |  |  |  |  |  |  |  |  |
| No vaccine | 161,802 (98.8) | 57,310 (99.4) | 104,402 (98.5) | Ref. |  | 5,054 (99.9) | 52,256 (99.4) | Ref. |  | 1,924 (99.9) | 55,228 (99.4) | Ref. |  |
| 1st dose 0-13 days | 265 (0.2) | 139 (0.2) | 126 (0.1) | -109.6% (-167%,-64.5%) | <0.001 | 1 (0.0) | 138 (0.3) | 78.1% (-56.7%,96.9%) | 0.001 | 1 (0.1) | 138 (0.2) | -65.8% (-1094.8%,77%) | 0.616 |
| 1st dose ≥14 days | 611 (0.4) | 103 (0.2) | 508 (0.5) | 61.3% (52.2%,68.7%) | <0.001 | 0 (0.0) | 103 (0.2) | 100% | - | 0 (0.0) | 103 (0.2) | 100% | - |
| 2nd dose 0-13 days | 189 (0.1) | 9 (0.0) | 180 (0.2) | 90.5% (81.4%,95.1%) | <0.001 | 0 (0.0) | 9 (0.0) | 100% | - | 0 (0.0) | 9 (0.0) | 100% | - |
| 2nd dose ≥14 days | 918 (0.6) | 90 (0.2) | 828 (0.8) | 79.3% (74.2%,83.3%) | <0.001 | 3 (0.1) | 87 (0.2) | 37.9% (-107.8%,81.4%) | <0.001 | 1 (0.1) | 89 (0.2) | 30.9% (-483.5%,91.8%) | 0.734 |
| **Ad26.CoV2.S (Johnson & Johnson/Janssen)** |  |  |  |  |  |  |  |  |  |  |  |  |  |
| No vaccine | 161,802 (99.9) | 57,310 (99.9) | 104,492 (99.9) | Ref. |  | 5,054 (100.0) | 52,256 (99.9) | Ref. |  | 1,924 (100.0) | 55,228 (99.9) | Ref. |  |
| 1st dose 0-13 days | 13 (0.0) | 6 (0.0) | 7 (0.0) | -65.9% (-395.4%,44.4%) | 0.371 | 0 (0.0) | 6 (0.0) | 100% | - | 0 (0.0) | 6 (0.0) | 100% | - |
| 1st dose ≥14 days | 113 (0.1) | 43 (0.1) | 70 (0.1) | -14.4% (-67.4%,21.9%) | 0.515 | 0 (0.0) | 43 (0.1) | 100% | - | 0 (0.0) | 43 (0.1) | 100% | - |
| 2nd dose ≥14 days | 5 (0.0) | 3 (0.0) | 2 (0.0) | -149.7% (-1406.9%,58.6%) | 0.316 | 0 (0.0) | 2 (0.0) | 100% | - | 0 (0.0) | 3 (0.0) | 100% | - |
| **BBIBP-CorV (Sinopharm)** |  |  |  |  |  |  |  |  |  |  |  |  |  |
| No vaccine | 161,802 (100) | 57,310 (100) | 104,492 (100) | Ref. |  | 5,054 (100.0) | 52,256 (100.0) | Ref. |  | 1,924 (100.0) | 55,228 (100.0) | Ref. |  |
| 1st dose 0-13 days | 1 (0.0) | 1 (0.0) | 0 (0.0) | 100% | - | 0 (0.0) | 1 (0.0) | 100% | - | 0 (0.0) | 1 (0.0) | 100% | - |
| 2nd dose 0-13 days | 1 (0.0) | 1 (0.0) | 0 (0.0) | 100% | - | 0 (0.0) | 1 (0.0) | 100% | - | 0 (0.0) | 1 (0.0) | 100% | - |
| 2nd dose ≥14 days | 6 (0.0) | 2 (0.0) | 4 (0.0) | 12.9% (-379%,84.2%) | 0.877 | 1 (0.0) | 1 (0.0) | -903.4% (-35078.3%,71.4%) | 0.847 | 0 (0.0) | 2 (0.0) | 100% | - |
| **NVX-CoV2373 (Novavax)** |  |  |  |  |  |  |  |  |  |  |  |  |  |
| No vaccine | 161,802 (100) | 57,310 (100) | 104,492 (100.0) | Ref. |  | 5,054 (100.0) | 52,256 (100.0) | Ref. |  | 1,924 (100.0) | 55,228 (100.0) | Ref. |  |
| 1st dose ≥14 days | 2 (0.0) | 2 (0.0) | 0 (0.0) | 0.00% | - | 0 (0.0) | 2 (0.0) | 100% | - | 0 (0.0) | 2 (0.0) | 100% | - |
| 2nd dose ≥14 days | 7 (0.01) | 0 (0.0) | 7 (0.0) | 100% | - | 0 (0.0) | 0 (0.0) | - | - | 0 (0.0) | 0 (0.0) | - | - |
| **Gam-COVID-Vac (Gamaleya’s Sputnik V)** |  |  |  |  |  |  |  |  |  |  |  |  |  |
| No vaccine | 161, 802 (100) | 57,310 (100.0) | 104,492 (100.0) | Ref. |  | 5,054 (100.0) | 52,256 (100.0) | Ref. |  | 1,924 (100.0) | 55,228 (99.9) | Ref. |  |
| 1st dose ≥14 days | 5 (0.0) | 0 (0.0) | 5 (0.0) | 100% | - | 0 (0.0) | 0 (0.0) | - | - | 0 (0.0) | 0 (0.0) | 100% | - |
| 2nd dose ≥14 days | 5 (0.0) | 2 (0.0) | 3 (0.0) | -12.9% (-579.2%,81.2%) | 0.890 | 0 (0.0) | 2 (0.0) | 100% | - | 0 (0.0) | 2 (0.003) | 100% | - |

**Table S1.** COVID-19 vaccines effectiveness in non-comorbid patients before Omicron.

OR – Odd ratios, OR adjusted for sex, age and tobacco smoking.
