# Supplementary material for: The impact of comorbidity status in COVID-19 vaccines effectiveness before and after SARS-CoV-2 omicron variant in northeastern Mexico: a retrospective multi-hospital study
Source: Front Public Health. 2024 Jun 12;12:1402527. doi: 10.3389/fpubh.2024.1402527 (PMC11199416; doi:10.3389/fpubh.2024.1402527)
Supplement: Supplementary file 1 [file Data_Sheet_1.ZIP › Table S13.docx]

**Table S13.** COVID-19 vaccines effectiveness in patients with more than two comorbidities before Omicron.

| **More than two comorbidities, before Omicron** | | | | | | | | | | | | | |
| --- | --- | --- | --- | --- | --- | --- | --- | --- | --- | --- | --- | --- | --- |
|  |  | COVID-19 infection | | | | Hospitalization | | | | Death | | | |
|  | Total | Yes | No | Effectiveness (95%CI) (Adjusted 1 – OR) | *p*-value | Yes | No | Effectiveness (95%CI) (Adjusted 1 – OR) | *p*-value | Yes | No | Effectiveness (95%CI) (Adjusted 1 – OR) | *p*-value |
| **BNT162b2 (Pfizer)** |  |  |  |  |  |  |  |  |  |  |  |  |  |
| No vaccine | 5,742 (92.0) | 1,840 (95.4) | 3,902 (90.4) | Ref. |  | 1,094 (96.2) | 746 (94.3) | Ref. |  | 646 (97.9) | 1,168 (94.3) | Ref. |  |
| 1st dose 0-13 days | 28 (0.4) | 6 (0.3) | 22 (0.5) | 43.2% (-40.5%,77%) | 0.221 | 3 (0.3) | 3 (0.4) | 22.5% (-312.4%,85.5%) | 0.766 | 0 (0.0) | 6 (0.5) | 100% (100%,100%) | 0.999 |
| 1st dose ≥14 days | 84 (1.3) | 19 (1.0) | 65 (1.5) | 37.4% (-4.7%,62.6%) | 0.074 | 13 (1.1) | 6 (0.8) | -132% (-542.9%,16.3%) | 0.106 | 5 (0.8) | 14 (1.1) | 9% (-166.3%,68.9%) | 0.863 |
| 2nd dose 0-13 days | 22 (0.4) | 5 (0.3) | 17 (0.4) | 36.8% (-71.9%,76.8%) | 0.369 | 0 (0.0) | 5 (0.6) | 100% | - | 0 (0.0) | 5 (0.4) | 100% | - |
| 2nd dose ≥14 days | 367 (5.9) | 58 (3.0) | 309 (7.2) | 60.5% (47.4%,70.3%) | <0.001 | 27 (2.4) | 31 (3.9) | 50.4% (13.7%,71.5%) | 0.013 | 9 (1.4) | 45 (3.6) | 69.7% (36.1%,85.7%) | 0.002 |
| **ChAdOx1 (AstraZeneca)** |  |  |  |  |  |  |  |  |  |  |  |  |  |
| No vaccine | 5,742 (92.1) | 1,840 (91.8) | 3,902 (92.2) | Ref. |  | 1,094 (94.7) | 746 (87.8) | Ref. |  | 646 (94.3) | 1,168 (90.7) | Ref. |  |
| 1st dose 0-13 days | 36 (0.6) | 16 (0.8) | 20 (0.5) | -73.8% (-236.6%,10.3%) | 0.101 | 4 (0.3) | 12 (1.4) | 60.3% (-28.1%,87.7%) | 0.122 | 3 (0.49 | 13 (1.09 | 20.6% (-189.9%,78.3%) | 0.727 |
| 1st dose ≥14 days | 147 (2.4) | 58 (2.9) | 89 (2.1) | -42% (-98.7%,-1.4%) | 0.041 | 17 (1.5) | 41 (4.8) | 64.7% (35.3%,80.8%) | 0.001 | 9 (1.3) | 46 (3.6) | 51.8% (-2.1%,77.3%) | 0.062 |
| 2nd dose 0-13 days | 31 (0.5) | 9 (0.4) | 22 (0.5) | 13% (-89.6%,60%) | 0.727 | 4 (0.3) | 5 (0.69 | 59.4% (-59.6%,89.7%) | 0.197 | 2 (0.3) | 7 (0.5) | 59.4% (-103%,91.9%) | 0.272 |
| 2nd dose ≥14 days | 284 (4.5) | 82 (4.1) | 199 (4.7) | 14.7% (-11.1%,34.5%) | 0.239 | 36 (3.1) | 46 (5.49 | 65.8% (45.2%,78.7%) | <0.001 | 25 (3.6) | 54 (4.2) | 40.2% (1%,63.9%) | 0.046 |
| **CoronaVac (Sinovac)** |  |  |  |  |  |  |  |  |  |  |  |  |  |
| No vaccine | 5,742 (96.7) | 1,840 (96.3) | 3,902 (96.8) | Ref. |  | 1,094 (98.4) | 746 (93.5) | Ref. |  | 646 (98.6) | 1,168 (95.1) | Ref. |  |
| 1st dose 0-13 days | 9 (0.2) | 2 (0.1) | 7 (0.2) | 40.7% (-186.1%,87.7%) | 0.515 | 1 (0.1) | 1 (0.1) | 12.4% (-1312%,94.6%) | 0.925 | 1 (0.2) | 1 (0.1) | -154.1% (-4007.8%,84.3%) | 0.511 |
| 1st dose ≥14 days | 42 (0.7) | 18 (0.9) | 24 (0.6) | -60.9% (-197.6%,13%) | 0.129 | 5 (0.4) | 13 (1.6) | 65.5% (-0.3%,88.1%) | 0.052 | 0 (0.0) | 18 (1.5) | 100% | - |
| 2nd dose 0-13 days | 13 (0.2) | 7 (0.4) | 6 (0.1) | -142% (-622.8%,19%) | 0.113 | 0 (0.0) | 7 (0.9) | 100% | - | 0 (0.0) | 7 (0.6) | 100% | - |
| 2nd dose ≥14 days | 135 (2.3) | 43 (2.3) | 92 (2.3) | 0.4% (-43.7%,31%) | 0.982 | 12 (1.1) | 31 (3.9) | 73.7% (47.2%,86.9%) | <0.001 | 8 (1.2) | 34 (2.8) | 53.6% (-3.3%,79.1%) | 0.061 |
| **Ad5-nCoV (CanSinoBIO)** |  |  |  |  |  |  |  |  |  |  |  |  |  |
| No vaccine | 5,742 (99.7) | 1,840 (99.7) | 3 ,902 (99.7) | Ref. |  | 1,094 (99.9) | 746 (99.5) | Ref. |  | 646 (99.8) | 1,168 (99.7) | Ref. |  |
| 1st dose 0-13 days | 1 (0.0) | 0 (0.0) | 1 (0.0) | 100% | - | 0 (0.0) | 0 (0.0) | - | - | 0 (0.0) | 0 (0.0) | - | - |
| 1st dose ≥14 days | 15 (0.3) | 5 (0.3) | 10 (0.3) | -10.9% (-225.8%,62.3%) | 0.851 | 4 (0.5) | 1 (0.1) | 67.7% (-201.5%,96.5%) | 0.321 | 1 (0.2) | 4 (0.3) | -3.5% (-374.5%,77.4%) | 0.984 |
| 2nd dose 0-13 days | 1 (0.0) | 0 (0.0) | 1 (0.0) | 100% | - | 0 (0.0) | 0 (0.0) | - | - | 0 (0.0) | 0 (0.0) | - | - |
| **mRNA-1273 (Moderna)** |  |  |  |  |  |  |  |  |  |  |  |  |  |
| No vaccine | 5,742 (99.7) | 1,840 (99.8) | 3,902 (99.6) | Ref. |  | 1,094 (100.0) | 746 (99.6) | Ref. |  | 646 (100.0) | 1,168 (99.7) | Ref. |  |
| 1st dose 0-13 days | 1 (0.0) | 1 (0.1) | 0 (0.0) | 0% | - | 0 (0.0) | 1 (0.1) | 100% | - | 0 (0.0) | 1 (0.1) | 100% | - |
| 1st dose ≥14 days | 4 (0.1) | 1 (0.1) | 3 (0.1) | 25.7% (-617.2%,92.3%) | 0.797 | 0 (0.0) | 1 (0.1) | 100% | - | 0 (0.0) | 1 (0.1) | 100% | - |
| 2nd dose 0-13 days | 3 (0.1) | 0 (0.0) | 3 (0.1) | 100% | - | 0 (0.0) | 0 (0.0) | - | - | 0 (0.0) | 0 (0.0) | - | - |
| 2nd dose ≥14 days | 11 (0.2) | 1 (0.1) | 10 (0.3) | 78.7% (-66.7%,97.3%) | 0.141 | 0 (0.0) | 1 (0.1) | 100% | - | 0 (0.0) | 1 (0.1) | 100% | - |

OR – Odd ratios, OR adjusted for sex, age, and tobacco smoking.
